# May 1,2-Dithiolane-4-carboxylic Acid and Its Derivatives Serve as a Specific Thioredoxin Reductase 1 Inhibitor?

**DOI:** 10.3390/molecules28186647

**Published:** 2023-09-15

**Authors:** Anna Nikitjuka, Kristaps Krims-Davis, Iveta Kaņepe-Lapsa, Melita Ozola, Raivis Žalubovskis

**Affiliations:** 1 Latvian Institute of Organic Synthesis, Aizkraukles 21, LV-1006 Riga, Latvia; kristaps.krims-davis@farm.osi.lv (K.K.-D.); iveta@farm.osi.lv (I.K.-L.); melita.videja@farm.osi.lv (M.O.); 2 Institute of Technology of Organic Chemistry, Faculty of Materials Science and Applied Chemistry, Riga Technical University, P. Valdena iela 3, LV-1048 Riga, Latvia

**Keywords:** asaparagusic acid, 1,2-dithiolane, TrxR, Michael acceptor

## Abstract

Thioredoxin reductase is an essential enzyme that plays a crucial role in maintaining cellular redox homeostasis by catalyzing the reduction of thioredoxin, which is involved in several vital cellular processes. The overexpression of TrxR is often associated with cancer development. A series of 1,2-dithiolane-4-carboxylic acid analogs were obtained to verify the selectivity of 1,2-dithiolane moiety toward TrxR. Asparagusic acid analogs and their bioisoters remain inactive toward TrxR, which proves the inability of the 1,2-dithiolane moiety to serve as a pharmacophore during the interaction with TrxR. It was found that the Michael acceptor functionality-containing analogs exhibit higher inhibitory effects against TrxR compared to other compounds of the series. The most potent representatives exhibited micromolar TrxR1 inhibition activity (IC_50_ varied from 5.3 to 186.0 μM) and were further examined with in vitro cell-based assays to assess the cytotoxic effects on various cancer cell lines and cell death mechanisms.

## 1. Introduction

Cancer as a disease depends on many factors involving genetic and epigenetic processes, thus interfering with essential cellular processes [1]. During the development of cancer, cells undergo various changes that allow undetermined proliferation and invasion of other organs [2]. Various endogenous and exogenous factors cause oxidative stress that leads to the activation of oncogenes, mutation of tumor suppression genes and metabolic changes in cancer cells [3]. That includes the formation of an imbalance between antioxidant capacity and the production of reactive oxygen species (ROS). Processes such as the upregulation of several antioxidant enzymes and components of glutathione and thioredoxin detoxification systems support the adaptation of cancer cells to oxidative stress [4,5]. Cancer cells able to control the increased ROS levels are associated with high metastatic possibility and resistance to traditional chemotherapy [6]. Therefore, targeting systems that control ROS in cancer cells represents a promising anticancer strategy.

The thioredoxin highly conserved system includes thioredoxin (Trx), thioredoxin reductases (TrxR) and NADPH. It represents a part of the response of cellular oxidative stress controlling intracellular ROS levels [7], where the key role of TrxR is to reduce Trx back to the dithiol form by abstraction electrons from NADPH [8].

**Figure 1:**
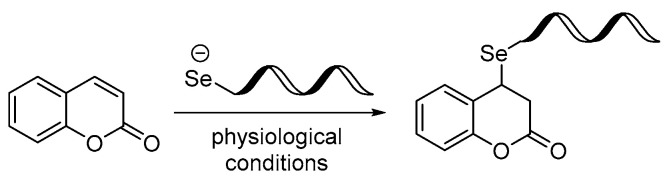
Representation of plausible reaction of TrxR1 selenocysteine residue (in its selenoate form) with Michael acceptor moiety (coumarin).

The thiol-dithiol interchange in the Trx system remains the predominant regulator in cell redox control and participates in antioxidant defense, selenium and nitric oxide metabolisms, etc. In mammalian cells, three TrxR isoforms are found: cytoplasmic and nuclear TrxR1, mitochondrial TrxR2 as well as less abundant TrxR3. TrxR1 is overexpressed in cancer cells, therefore it is a promising target for the development of novel anticancer agents [9]. The redox-active site on the C-terminal of mammalian TrxR1 possesses a distinctive selenocysteine (Sec) residue, which is exposed on the surface of the enzyme in its reduced form [10]. Moreover, under physiological conditions Sec is presented in its selenoate form, making the enzyme very sensitive to covalent inhibition by small electrophilic compounds, such as Michael acceptors, i.e., C=C bonds directly attached to an electron-withdrawing group (Figure 1) [11,12,13,14]. On the other hand, TrxR1 can also be targeted by compounds that mimic its natural substrates with disulfide moiety, where the participation of the second TrxR1 redox-active site, containing a homological sequence of Cys-Val-Asn-Val-Gly-Cys-, is not excluded [15]. The investigation of cyclic disulfide analogs, such as asparagusic acid [16,17] and its analogs [18], remains an attractive and less-investigated concept in the design of potential TrxR inhibitors.

Several studies have demonstrated the potential of specific 1,2-dithiolane-containing compounds to serve as highly selective probes and inhibitors of TrxR [19,20]. However, a certain level of discrepancy occurs in the recently published studies [21] showing that 1,2-dithiolane moiety can be nonspecifically reduced by a wide range of cell-presented thiols. We aim to gain a better understanding of whether 1,2-dithiolane-4-carboxilic acid analogs, starting from the most primitive representative, asparagusic acid and its bioisosters, possess an increased activity toward TrxR. Moreover, among the presented derivatives, we have combined both the Michael acceptors (coumarin moiety) and the 1,2-dithiolane moiety (asparagusic acid moiety) in the same molecule. We also provide a structure-activity relationship (SAR) analysis regarding the inhibitory potential toward TrxR1. To better guide future research on TrxR inhibitors containing asparagusic acid residue, the characterization of the most potent compounds as anticancer agents and the evaluation of cell death mechanisms are performed.

## 2. Results and Discussion

### 2.1. Synthesis of the Target Compounds

**Scheme 1:**
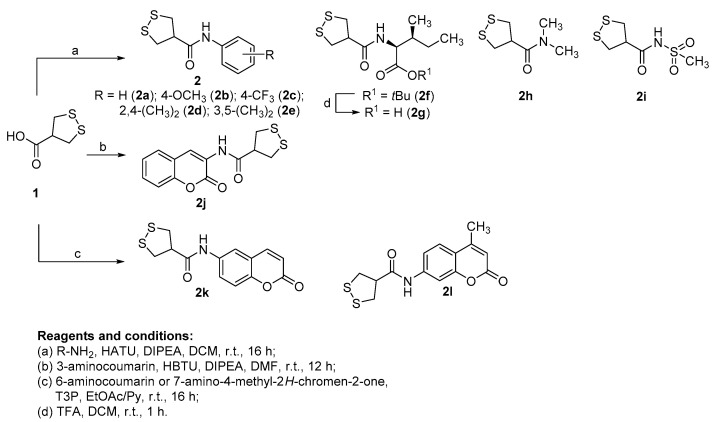
Synthetic conditions for the synthesis of analogs **2a**–**2l**.

The construction of a library containing asparagusic acid moiety is presented in Scheme 1. Thus, for the efficient synthesis, different reaction conditions were applied and, as a result, the target compounds were obtained in moderate to good yields (Scheme 1). 1,2-Dithiolane-4-carboxylic acid (**1**, asparagusic acid) was obtained following a known procedure with the formation of dihydroasparagusic acid (DHAA) as the main intermediate from dibromide precursor followed by cyclization [22].

Asparagusic acid **1** was coupled with available amine derivatives using one of three approaches, a, b or c, to furnish the target compounds **2a**–**l** (Scheme 1). Thus, the synthesis of **2a**–**e**, dimethylamide **2f** and the bioisosteric sulfonamide analog **2g** was performed in the presence of hexafluorophosphate azabenzotriazole tetramethyl uronium (HATU) and *N*,*N*-diisopropylethylamine (DIPEA) (conditions a). Subsequently, the analogs **2j**, **2k** and **2l** were obtained, applying a hexafluorophosphate benzotriazole tetramethyl uronium (HBTU) and propylphosphonic anhydride (T3P) solution in EtOAc (conditions b and c, respectively). The obtained analogs were stable during purification and storage. Data on synthesized novel asparagusic acid-containing compounds were presented in the SI. NMR (^1^H NMR and ^13^C NMR), high-resolution mass spectrometry (HRMS) analyses reliably confirm the structure of the obtained compounds.

### 2.2. TrxR1 Inhibitory Activity and SAR Evaluation

First, we examined the interaction of compounds **2a**–**l** with TrxR1. The TrxR1 inhibitory activity was evaluated with recombinant rat TrxR1 using a 5,5′-dithiol-bis-2-nitrobenzoic acid (DTNB) reduction assay at an initial concentration of 200 μM of the target compound (Table 1).

The preliminary TrxR1 inhibitory assay undoubtedly revealed that the presence of a lone 1,2-dithiolane moiety is insufficient for considerable activity towards TrxR1. We were very upset to discover that the inhibitory potency of compounds **2a**–**e**, **2f**, **2h**, **2i** and **2l** remained less than 50% at 200 μM concentration. Among the aromatic analogs obtained, neither change of aryl substituents and incorporation of electron-donating (compounds **2b**, **2d** and **2e**, Scheme 1) or electron-withdrawing (compound **2c**, Scheme 1) substituents at C-4 position of the aromatic system did not influence the inhibitory activity of TrxR1. Similarly, asparagusic acid (**1**) itself, dimethylamino analog (**2h**) and bioisosteric sulfonamide (**2i**), practically did not inhibit the TrxR1 and cannot serve as selective, small-size inhibitors of TrxR1. Despite several reports on the remarkable selectivity of the 1,2-dithiolane moiety toward TrxR [23], our observations support the idea that the 1,2-dithiolane cycle has no impact on TrxR1 activity, and without an additional TrxR-selective fragment, such as Michael acceptor [13,14], it remains inactive. Thus, the coumarin analogs **2j** and **2k** were the most active among the series (Scheme 1). We perceive that inhibitory activity towards TrxR1 is strongly associated with the presence of an accessible and activated double bond at the coumarin core that can interact with the TrxR1 Sec residue. The presence of the electron-withdrawing group at C-3 of coumarin, as in the case of **2j,** results in the elevated electrophilicity of 3,4-coumarin double bond, which leads to five-fold higher TrxR1 activity of analog **2j** than that of analog **2k**. Notably, we observed the absence of inhibitory activity of analog **2l**, where a plausible 1,4-addition of nucleophilic Sec-residue is complicated and might be disfavored in contrast to *N*-(2-oxo-2H-chromen-6-yl)-1,2-dithiolane-4-carboxamide derivative **2k** due to the presence of the methyl group at C-4 of coumarin core. This observation provides reliable evidence for the crucial role of Michael acceptor moiety, rather than that of 1,2-dithiolane moiety, in determining TrxR1 activity.

Based on the preliminary TrxR1 inhibitory potential, only three of the obtained compounds, **2g**, **2j** and **2k,** were efficient at 200 μM concentration and became a focus of our study. They were further tested in dose-dependent TrxR1 inhibition assays and validated at different concentrations (2 to 200 μM) to determine the IC_50_ values. Thus, the selected leaders displayed inhibition potency for the TrxR1 enzyme at a micromolar range with the highest activity of compound **2j**, for which the IC_50_ value towards the TrxR1 enzyme was 5.3 ± 0.4 µM.

### 2.3. Cytotoxic Effect

Since three of the obtained analogs, **2g**, **2j** and **2k,** displayed inhibitory potency in the enzymatic assay (Table 1), this encouraged us to further test these compounds for their cytotoxic effects on cancerous and normal cells. Interestingly, there was no direct correlation between the TrxR1 inhibitory potency of the tested compounds and their cytotoxic effects. As expected, the compound **2g** did not show significant cytotoxic activity. The compound **2j** with the lowest IC_50_ value towards TrxR1 exhibited low cytotoxicity on the cell lines in the tested concentration range. Moreover, the cytotoxic effect was even more pronounced on non-cancerous HEK293 cells. On the contrary, compound **2k,** which showed moderate activity in the enzymatic assay, exhibited its cytotoxic effects in all tested cell lines in sub-micromolar concentrations. Unfortunately, compound **2k** did not show any selectivity towards cancerous cells. This means that future research must be targeted at improving the selectivity of the newly synthesized compounds.

### 2.4. Cell Death Assay

**Figure 2:**
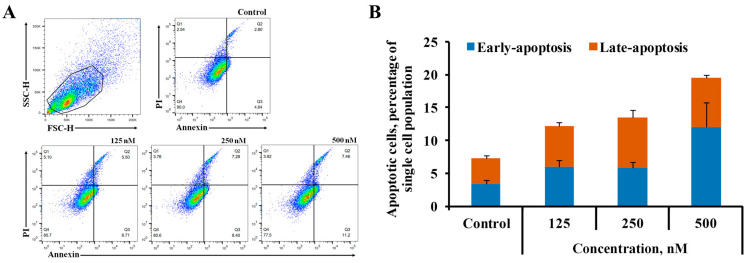
Representative plots from flow cytometry analysis of cell death after treatment with compound **2k,** up to 500 nM final concentrations for 20 h in 4T1 cells (**A**). Effects of compound **2k** on induction of apoptosis, presented as percentage of single cell population (marked with a black contour line in panel (**A**)) (**B**) The data are presented as the mean ± SEM of three independent experiments performed in duplicate.

To evaluate the mechanisms of cell death induction after incubation with compound **2k**, we used Annexin V and PI staining following an analysis using flow cytometry (Figure 2). After incubation with **2k** at different concentrations up to 500 nM for 20 h, we observed a significant increase in the apoptotic cell count, compared to untreated control cells. Moreover, the increase in the apoptotic cell count was dose-dependent, reaching 19.3% (early apoptosis—12% and late apoptosis—7.3%) in the highest concentration tested. At the same time, the count of necrotic cells did not change significantly, indicating an induction of apoptotic cell death. These results indicate that the test compound **2k** instigates programmed cell death mechanisms, which could be beneficial in terms of cancerous cells, as apoptotic cell death is not associated with the activation of severe inflammation.

## 3. Materials and Methods

### 3.1. General

Unless otherwise noted, all of the reagents were obtained from commercial sources and used without further purification. All of the reactions were monitored by using thin-layer chromatography (TLC) on Merck 60 F254 pre-coated silica gel plates and were visualized using a UV lamp stained with KMnO_4_. Column chromatography was performed using Kieselgel silica gel (35–70 and 60–200 μm). Reactions were performed using standard glassware according to the literature sources. ^1^H, ^13^C, ^19^F spectra were recorded on a 400 MHz Bruker spectrometer using residual solvent peak as a reference (CDCl_3_, Acetone-*d*_6_, MeOH-*d*_4_ and Acetonitrile*-d*_3_). Compounds for HRMS were analyzed by positive mode electrospray ionization (ESI) using a Waters Synapt G2-Si mass spectrometer. Importantly, all the title compounds were new.

#### 3.1.1. General Synthetic Procedure for the Target Compounds **2a**–**i** (Conditions a)

To the solution of asparagusic acid (**1**) (0.5 mmol, 1 equiv) and aniline derivative (1 equiv) in the mixture of DCM/DMF (10 mL, 10:1), HATU (1.2 equiv) was added in one portion, followed by the addition of DIPEA (1.2 equiv). The reaction mixture was stirred under an Ar atmosphere at r.t. overnight (16 h). The reaction mixture was treated with water (2 × 20 mL), and the product was extracted with DCM (10 mL) and washed with an additional amount of water (20 mL). The combined organic phase was dried over Na_2_SO_4_, filtrated and evaporated. The residue was chromatographed on a silica gel column with ethyl acetate/petroleum ether (1/2) to provide the target compound.

*N-Phenyl-1,2-dithiolane-4-carboxamide* (**2a**). Yield: 37 mg (32%); colorless solid; m.p. = 112 °C (dec.). ^1^H NMR (CDCl_3_, 400 MHz), *δ*, ppm: 7.60 (br.s, 1H); 7.53–7.46 (m, 2H); 7.37–7.30 (m, 2H); 7.17–7.11 (m, 1H); 3.53–3.42 (m, 4H); 3.42–3.33 (m, 1H). ^13^C NMR (CDCl_3_, 100 MHz), *δ*, ppm: 170.1; 137.5; 129.3; 124.9; 120.1; 53.4; 43.2. HRMS (*m*/*z*) calcd for C_10_H_12_NOS_2_ ([M + H]^+^) 226.0360. Found: 226.0356.

*N-(4-Methoxyphenyl)-1,2-dithiolane-4-carboxamide* (**2b**). Yield 49 mg (38%); colorless solid; m.p. = 138–140 °C. ^1^H NMR (CDCl_3_, 400 MHz), *δ*, ppm: 7.53 (br.s, 1H); 7.42–7.36 (m, 2H); 6.89–6.83 (m, 2H); 3.79 (s, 3H); 3.52–3.39 (m, 4H); 3.39–3.29 (m, 1H).^13^C NMR (CDCl_3_, 100 MHz), *δ*, ppm: 169.9; 156.9; 130.5; 122.1; 114.4; 55.6; 53.3; 43.2. HRMS (*m*/*z*) calcd for C_11_H_14_NO_2_S_2_ ([M + H]^+^) 256.0466. Found: 256.0471.

*N-(4-(Trifluoromethyl)phenyl)-1,2-dithiolane-4-carboxamide* (**2c**). Yield 42 mg (29%); colorless solid; m.p. = 179–181 °C. ^1^H NMR (CDCl_3_, 400 MHz), *δ*, ppm: 7.82 (s, 1H); 7.70–7.50 (m, 4H); 3.57–3.35 (m, 5H). ^13^C NMR (CDCl_3_, 100 MHz), *δ*, ppm: 170.5; 140.6; 126.5 (q, *J* = 3.8 Hz); 124.0 (q, *J* = 272.2 Hz); 119.7; 53.2; 43.2. ^18^F NMR (CDCl_3_, 100 MHz), *δ*, ppm.: −66.2. HRMS (*m*/*z*) calcd for C_11_H_11_F_3_NOS_2_ ([M + H]^+^) 294.0234. Found: 294.0240.

*N-(2,4-dimethoxyphenyl)-1,2-dithiolane-4-carboxamide* (**2d**). Yield 54 mg (38%); colorless solid; m.p. = 116 °C (dec.).^1^H NMR (400 MHz, CDCl_3_), *δ*, ppm: 8.23—8.15 (m, 1H); 7.83 (brs, 1H); 6.46 (d, *J* = 10.4 Hz, 2H); 3.86 (s, 3H); 3.79 (s, 3H); 3.51–3.43 (m, 4H); 3.36–3.28 (m, 1H). ^13^C NMR (101 MHz, CDCl_3_) δ, ppm: 169.3; 156.9; 149.4; 120.9; 103.9; 98.8; 55.9; 55.7; 53.8; 43.1. HRMS (*m*/*z*) calcd for C_12_H_16_NO_3_S_2_ ([M + H]^+^) 286.0572. Found: 286.0576.

*N-(3,5-Dimethoxyphenyl)-1,2-dithiolane-4-carboxamide* (**2e**). Yield 50 mg (30%); colorless solid; m.p. = 118–121 °C. ^1^H NMR (400 MHz, Acetone) *δ*, ppm: 9.25 (s, 1H); 6.90 (d, *J* = 2.2 Hz, 2H); 6.24 (t, *J* = 2.3 Hz, 1H); 3.75 (s, 6H); 3.50–3.44 (m, 2H); 3.30–3.26 (m, 2H); 3.13–3.07 (m, 1H). ^13^C NMR (101 MHz, Acetone) δ 162.1; 141.8; 98.7; 96.7; 55.7; 37.5. HRMS (*m*/*z*) calcd for C_12_H_16_NO_3_S_2_ ([M + H]^+^) 286.0572. Found: 286.0578.

*tButyl 2-(1,2-dithiolane-4-carboxamido)-3-methylpentanoate* (**2f**). Yield 53 mg (33%); colorless amorphous solid. ^1^H NMR (CDCl_3_, 400 MHz), *δ*, ppm: 6.30 (d, *J* = 8.5 Hz, 1H); 4.48 (dd, *J* = 8.4, 4.4 Hz, 1H); 3.43–3.32 (m, 4H); 3.31–3.21 (m, 1H); 1.89 (dqt, *J* = 9.1, 6.8, 4.6 Hz, 1H); 1.47 (s, 9H); 1.36–1.09 (m, 2H, partially overlapped with O*t*Bu signal); 0.96–0.83 (m, 6H). ^13^C NMR (CDCl_3_, 100 MHz), *δ*, ppm: 171.5; 170.9; 82.5; 57.0; 52.5; 43.0; 42.9; 38.2; 28.2; 25.5; 15.6; 11.9. HRMS (*m*/*z*) calcd for C_14_H_24_NO_3_S_2_ ([M − H]^+^) 318.1198. Found: 318.1193. ^13^C NMR (101 MHz, CDCl_3_) δ.

*2-(1,2-Dithiolane-4-carboxamido)-3-methylpentanoic acid* (**2g**). The obtained product **2f** (50 mg, 16 mmol) was dissolved in DCM (5 mL), and TFA was added in one portion (1 mL). Continue to stir at r.t. until full starting material consumption (TLC-control reaction, eluent EtOAc). The reaction mixture was evaporated and the product was recrystallized from Et_2_O. Yield: 28 mg (65%); white solid; m.p. = 135 °C (dec.). ^1^H NMR (MeOH-*d*_4_, 400 MHz), *δ*, ppm: 4.50–4.44 (m, *J* = 5.9, 3.0 Hz, 1H); 3.28–2.76 (m, 5H); 2.00–1.84 (m, 1H); 1.63–1.45 (m, 1H); 1.36–1.23 (m, 2H); 1.03–0.85 (m, 6H). ^13^C NMR (MeOH-*d*_4_, 100 MHz), *δ*, ppm: 173.3; 163.5; 56.9; 37.1; 35.6; 30.3; 25.1; 24.9; 15.0; 10.6. HRMS (*m*/*z*) calcd for C_10_H_16_NO_3_S_2_ ([M − H]^+^) 262.0572. Found: 262.0580.

*N,N-Dimethyl-1,2-dithiolane-4-carboxamide* (**2h**). Yield: 36 mg (41%); colorless solid; m.p. = 134–138 °C. ^1^H NMR (CDCl_3_, 400 MHz), *δ*, ppm: 3.47–3.30 (m, 5H), 3.11 (s, 3H), 2.98 (s, 3H). ^13^C NMR (CDCl_3_, 100 MHz), *δ*, ppm: 171.1; 48.7; 42.1; 37.4; 36.0. HRMS (*m*/*z*) calcd for C_6_H_12_NOS_2_ ([M + H]^+^) 178.0360. Found: 178.0365.

*N-(Methylsulfonyl)-1,2-dithiolane-4-carboxamide* (**2i**). Yield: 32 mg (28%); colorless solid; m.p. = 141–142.6 °C. ^1^H NMR (MeOH-*d*_4_, 400 MHz), *δ*, ppm: δ 3.41–3.35 (m, 4H); 3.33 (dd, *J* = 3.1, 1.6 Hz, 1H); 3.25 (s, 3H). ^13^C NMR (MeOH-*d*_4_, 100 MHz), *δ*, ppm: 171.7; 51.4; 41.5; 39.9. HRMS (*m*/*z*) calcd for C_5_H_8_NO_3_S_3_ ([M + H]^+^) 225.9666. Found: 225.9671.

#### 3.1.2. Synthesis of *N*-(2-Oxo-2H-chromen-3-yl)-1,2-dithiolane-4-carboxamide (**2j**) (Conditions b)

To the solution of asparagusic acid (**1**) (0.100 g, 0.66 mmol, 1 equiv) and 6-aminocoumarin (0.107 g, 0.66 mmol, 1 equiv), DMF (2 mL) was added to DIPEA (0.23 mL, 1.33 mmol, 2 equiv.) and HBTU (0.265 g, 0.69 mmol, 1.05 equiv) in one portion. The reaction mixture was stirred overnight at r.t., treated with water (10 mL) and the target compound was extracted with EtOAc (2 × 10 mL). Organic phase was combined, washed with H_2_O (20 mL) and brine (20 mL), dried over Na_2_SO_4_, filtrated and evaporated. The residue was purified by flash chromatography on silica gel, eluent EtOAc/petroleum ether (1/1). Yield: 86 mg (44%); light yellow amorphous solid. ^1^H NMR (400 MHz, CDCl_3_) *δ*, ppm: 8.71 (s, 1H); 8.02 (s, 1H); 7.56–7.42 (m, 2H); 7.38–7.27 (m, 2H); 4.19 (tt, *J* = 9.1, 8.1 Hz, 1H); 3.76–3.66 (m, 2H); 3.34–3.25 (m, 2H). ^13^C NMR (101 MHz, CDCl_3_) *δ*, ppm: 171.4; 158.9; 150.1; 130.1; 128.1; 125.5; 124.1; 123.8; 119.8; 116.6; 45.0; 27.2.

#### 3.1.3. Synthesis of *N*-(2-Oxo-2H-chromen-6-yl)-1,2-dithiolane-4-carboxamide (**2k**) and *N*-(4-Methyl-2-oxo-2H-chromen-7-yl)-1,2-dithiolane-4-carboxamide (**2l**) (Conditions c)

To the solution of asparagusic acid **1** (0.26 g, 1.6 mmol, 2 equiv) in the mixture of dry EtOAc/pyridine (4 mL/2 mL) was added aminocoumarin (0.135 mg, 0.8 mmol, 1 equiv). The heterogeneous mixture was cooled in an ice bath during the addition of T3P solution (50% in EtOAc) (1 mL, 1.6 mmol, 2 equiv) dropwise to avoid overheating. The resulting homogeneous solution was stirred at r.t. 1 h until the full conversion of the starting material (TLC-control reaction). The solution of HCl (1 M) (12 mL) was added to the reaction mixture, and the product was extracted with EtOAc (2 × 15 mL). The combined organic phases were washed with H_2_O (20 mL), dried over Na_2_SO_4_, filtrated and evaporated. The obtained residue was purified by flash chromatography, eluent EtOAc/petroleum ether (1/1) to EtOAc (100%).

*N-(2-oxo-2H-chromen-6-yl)-1,2-dithiolane-4-carboxamide* (**2k**). Yield: 114 mg (46%); m.p. = 132 °C (dec.). ^1^H NMR (400 MHz, Acetone/CD_3_CN), δ, 8.72 (s, 1H); 7.98 (d, *J* = 2.5 Hz, 1H); 7.85 (d, *J* = 9.7 Hz, 1H); 7.71–7.53 (m, 1H); 7.41–7.17 (m, 1H); 6.39 (d, *J* = 9.6 Hz, 1H); 3.76–3.03 (m, 5H). ^13^C NMR (101 MHz, Acetone/CD_3_CN) δ, ppm: 170.9; 161.3; 151.3; 144.6; 136.1; 124.4; 119.2; 117.8; 117.7; 53.4; 43.4; 27.6. HRMS (*m*/*z*) calcd for C_13_H_12_NO_3_S_2_ ([M + H]^+^) 294.0259. Found: 294.0261.

*N-(4-methyl-2-oxo-2H-chromen-7-yl)-1,2-dithiolane-4-carboxamide* (**2l**). Yield: 98 mg (34%); m.p. = 146 °C (dec.). ^1^H NMR (400 MHz, DMSO-*d*_6_), δ, 7.80 (brs, 1H); 7.40 (d, *J* = 8.6 Hz, 1H); 6.56 (dd, *J* = 8.6, 2.2 Hz, 1H); 6.40 (d, *J* = 2.1 Hz, 1H); 5.89 (d, *J* = 1.4 Hz, 1H); 3.08 (qd, *J* = 7.3, 4.8 Hz, 1H), 2.29 (d, *J* = 1.2 Hz, 3H). ^13^C NMR (101 MHz, DMSO-*d*_6_) δ 166.8; 160.7; 155.5; 153.7; 153.0; 126.2; 111.2; 108.9; 107.5; 98.6; 48.6; 45.6; 18.0. (HRMS (*m*/*z*) calcd for C_14_H_14_NO_3_S_2_ ([M + H]^+^) 308.0415. Found: 308.0422.

### 3.2. In Vitro Biological Testing

#### 3.2.1. Cell Culture Conditions

All cell lines used in the experiments were purchased from the American Type Culture Collection (ATCC, Manassas, VA, USA) and grown in the cell media according to the vendor’s instructions. DMEM, MEM of RPMI cell medium, was supplemented with 10% (*v*/*v*) FBS, streptomycin (100 µg/mL) and penicillin (100 U/mL). The cell culture was incubated under standard culture conditions at 37 °C with 5% CO_2_ in a humidified atmosphere and sub-cultured every 2 to 3 days. To ensure the maintained cell phenotype, the cells were used for experiments up to the 10th passage [24]. All cell lines were regularly controlled for mycoplasma contamination using a MycoProbe mycoplasm detection kit (R & D Systems, Inc., Minneapolis, MN, USA). Possible bacterial contamination was monitored by visual inspection before each cell passage and before the experiments.

#### 3.2.2. Cell Cytotoxicity Assay

For the cell cytotoxicity assay, HEK293, Hep G2, HeLa, 4T1, MDA MB 231, MDA MB 453, A549 and HT1080 cell lines were used. Each cell line was seeded in a 96-well plate at a density of 1 × 10^5^ cells per milliliter, total volume of 100 μL in each well. After seeding, cells were left to rest for 6 h. Various concentrations of test compounds dissolved in DMSO were added to the cell media and incubated for 48 h; vehicle solution was added to control cells to ensure the same DMSO end concentration of 1% in all wells. After the incubation, cell viability was assessed by MTT assay [25]. Briefly, 100 μL of MTT solution was added to all wells (end concentration 1 mg/mL) and incubated for 2 h at +37 °C. After incubation, the MTT solution was discarded and 100 μL of isopropanol was added to dissolve the sediment. Absorption was measured at 570 and 650 nm using a Hidex Sense microplate reader. The IC_50_ values were calculated with GraphPad Prism 8.0 (GraphPad, Inc., San Diego, CA, USA).

#### 3.2.3. Cell Death Assay Using Flow Cytometry Analysis

4T1 cells were seeded in 24-well plates at a density of 1 × 10^5^ cells/mL in a RPMI cell medium supplemented with 10% (*v*/*v*) FBS, streptomycin (100 µg/mL) and penicillin (100 U/mL). The cells were then allowed to rest and adhere to the plate for 5 h. Subsequently, the cells were treated with compound **2k** with final concentrations up to 500 nM for a 20 h time period.

To detect the mechanisms of 4T1 cell death, we performed double staining using Annexin-V (BioLegend, San Diego, CA, USA) and PI (Thermo Fisher Scientific, P1304MP, Eugene, OR, USA) [26]. In summary, after incubation with the test compounds, cells were harvested using trypsin, washed with 1× PBS and resuspended in 100 µL of staining buffer with Annexin V conjugated to APC (1:200) and PI (end concentration 2 μg/mL). The samples were incubated for 20 min in the dark at r.t. and then analyzed by flow cytometry using the BD FACS Melody Cell Sorter (BD Biosciences, San Jose, CA, USA).

#### 3.2.4. Colorimetric Detection of Rat TrxR1 Activity

Experiments were performed in 96-well plates with 100 µL of 100 mM potassium phosphate, pH 7.0, containing 1 mM EDTA, 0.1 mg/mL BSA and 0.2 mM NADPH (pH 7.5). A 5 nM concentration of recombinant active rat TrxR1 (IMCO Corporation Ltd. AB, Sweden) was incubated with different concentrations of tested compounds (**2a**–**2l**) for 15 min on a plate shaker at r.t. [27]. The concentration range of compounds for IC_50_ determination was 200–0.5 µM. For the control experiment, the appropriate amount of DMSO was used instead of the tested compound. After incubation, DTNB solution with a final concentration of 5 mM was added to the wells. Enzyme kinetics was monitored on a TECAN Infinite M1000 PRO microplate reader (Tecan Austria GmbH, Grödig, Austria) recording the changes (increase) in absorbance at 412 nm every minute for 15 min. Compounds were tested in triplicate. The reaction rate in OD/min was calculated. The activity of the positive control (no inhibitor) is 100%. The IC_50_ values were calculated with GraphPad Prism 8.0 using the nonlinear regression, variable slope (for the full description of IC_50,_ the calculation is presented in the Supplementary Materials).

## 4. Conclusions

Herein, we present the synthesis of 1,2-dithiolane-4-carboxylic acid moiety-containing library as possible TrxR1 inhibitors. Most of the target compounds obtained remained inactive towards TrxR1 and did not exhibit the desired inhibition of TrxR1. We conclude that the presence of lone 1,2-dithiolane moiety is not sufficient to provide TrxR1-specific inhibitors. It was confirmed that the inhibitory activity towards TrxR1 is strongly associated with the presence of an accessible Michael acceptor moiety capable of interaction with TrxR1 Sec and/or Cys residues. Thus, the combination of 1,2-dithiolane and Michael acceptor moieties led to the compound **2k** with a high TrxR1 inhibition potency along with promising cytotoxicity in various cancer cell lines.

The presence of 1,2-dithiolane alone does not provide TrxR1 selective inhibition, while in combination with activated double-bond, this type of compound has the potential to be developed into anticancer agents.

## Figures and Tables

**Table 1:** TrxR1 inhibitory activity of three asparagusic acid-containing inhibitors **2g, j, k** and their cytotoxic effect in cancer and normal cells.

Compound	% TrxR1 Activity Inhibition at 200 µM	IC_50_ ± SD, µM								
		TrxR1	HEK293	HepG2	HeLa	4T1	MDA MB 231	MDA MB 435	A549	HT1080
2g	50	186.4± 14.9	1.74 ± 0.57	>2	>2	>2	>2	>2	>2	>2
2j	100	5.3 ± 0.4	0.53 ± 0.23	1.56 ± 0.39	>2	>2	>2	>2	>2	1.80 ± 0.56
2k	89	36.3 ± 2.9	0.41 ± 0.12	0.29 ± 0.08	0.17 ± 0.03	0.24 ± 0.06	0.62 ± 0.14	0.25 ± 0.05	0.28 ± 0.04	0.31 ± 0.07

The data are presented as the mean and SD values of three independent experiments in three technical replicates.

## Data Availability

The data presented in this study are available in this article or the Supplementary Materials.

## Supplementary Materials

The following supporting information can be downloaded at: https://www.mdpi.com/article/10.3390/molecules28186647/s1.
