# Supplementary material for: May 1,2-Dithiolane-4-carboxylic Acid and Its Derivatives Serve as a Specific Thioredoxin Reductase 1 Inhibitor?
Source: Molecules. 2023 Sep 15;28(18):6647. doi: 10.3390/molecules28186647 (PMC10535816; doi:10.3390/molecules28186647)
Supplement: Supplementary file 1 [file molecules-28-06647-s001.zip › molecules-2541085-supplementary.pdf]

May 1,2-dithiolane-4-carboxylic acid and its derivatives serve as a specific thioredoxin reductase 1 inhibitor?

Nikitjuka Anna[1,*], Krims-Davis Kristaps[1], Kaņepe Iveta[1], Ozola Melita[1], Žalubovskis Raivis[1,2,*]

[1]Latvian Institute of Organic Synthesis, Aizkraukles 21, LV-1006, Riga, Latvia
[2]Institute of Technology of Organic Chemistry, Faculty of Materials Science and Applied Chemistry, Riga Technical University, P. Valdena iela 3, LV-1048 Riga, Latvia

*Corresponding authors: Anna Ņikitjuka, Raivis Žalubovskis

anna@osi.lv; raivis@osi.lv

Table of Contents

N-Phenyl-1,2-dithiolane-4-carboxamide (2a) ($CDCl_3$) 3

N-(4-Methoxyphenyl)-1,2-dithiolane-4-carboxamide (2b) ($CDCl_3$) 5

N-(4-(Trifluoromethyl)phenyl)-1,2-dithiolane-4-carboxamide (2c) ($CDCl_3$) 7

N-(2,4-dimethoxyphenyl)-1,2-dithiolane-4-carboxamide (2d) ($CDCl_3$) 9

N-(3,5-Dimethoxyphenyl)-1,2-dithiolane-4-carboxamide (2e) (Acetone-d6) 11

*t*Butyl 2-(1,2-dithiolane-4-carboxamido)-3-methylpentanoate (2f) ($CDCl_3$) 13

2-(1,2-Dithiolane-4-carboxamido)-3-methylpentanoic acid (2g) (MeOH-d4) 15

N,*N*-Dimethyl-1,2-dithiolane-4-carboxamide (2h) 17

N-(Methylsulfonyl)-1,2-dithiolane-4-carboxamide (2i) (MeOH-d4) 19

N-(2-Oxo-2H-chromen-6-yl)-1,2-dithiolane-4-carboxamide (2j) 21

N-(2-oxo-2H-chromen-3-yl)-1,2-dithiolane-4-carboxamide (2k) (CD_3CN/Acetone, low solubility) 22

N-(4-methyl-2-oxo-2H-chromen-7-yl)-1,2-dithiolane-4-carboxamide (2l, 1,2-dithiolane signal is overlapped with residue water signal) 24

Determination of the Reaction Rate and IC_{50} of colorimetric TrxR1 enzymatic assays 26

N-Phenyl-1,2-dithiolane-4-carboxamide (2a) ($CDCl_3$)

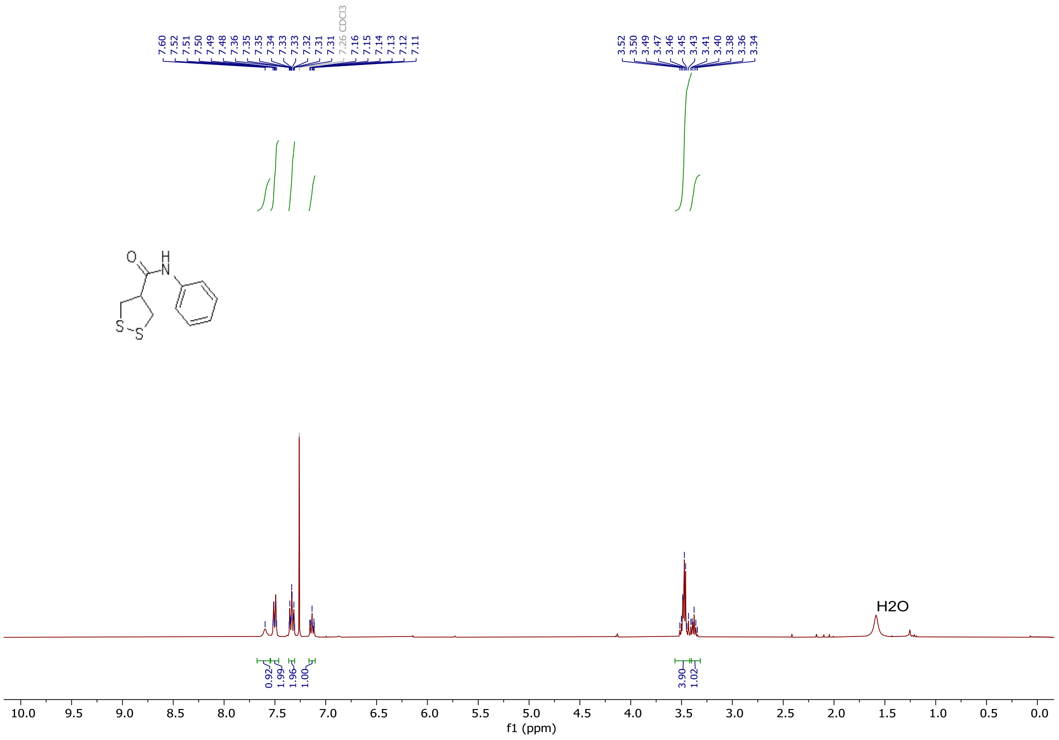

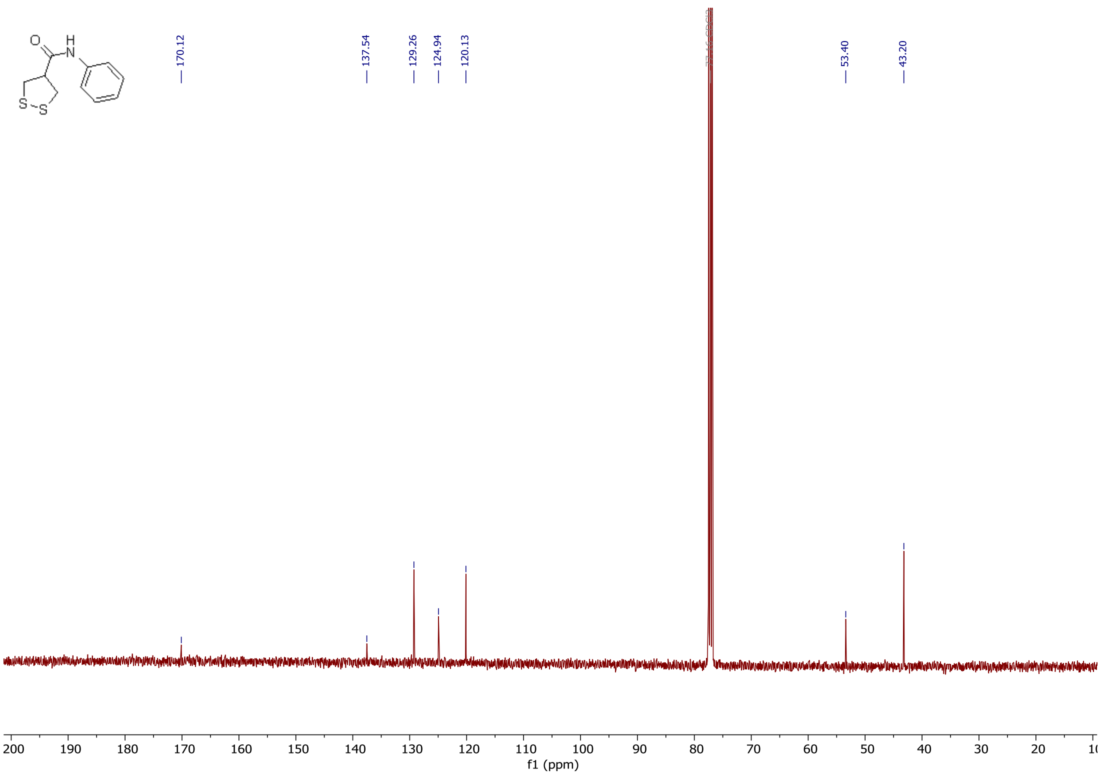

Elemental Composition Report

Tolerance = 5.0 PPM / DBE: min = -1.5, max = 200.0
Element prediction: Off
Number of isotope peaks used for i-FIT = 5

Monoisotopic Mass, Even Electron Ions
138 formula(e) evaluated with 1 results within limits (all results (up to 1000) for each mass)
Elements Used:
C: 0-50 H: 0-100 N: 0-3 O: 0-3 S: 0-2

2883 Nikitjuka **2a**

HRMS_2022_04_528 689 (1.970) Cm (689:699-(663:675+723:729))

OSI/FOKL-MS
Synapt G2-Si
1: TOF MS ES+
4.95e+005

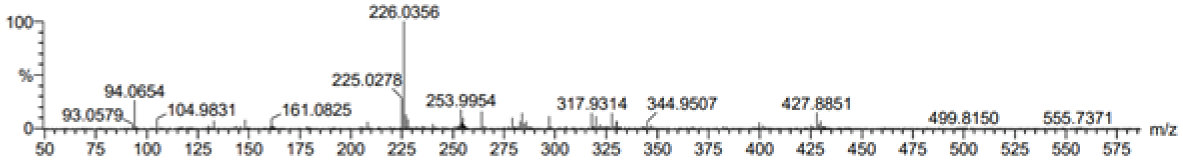

Minimum:	80.00				-1.5				
Maximum:	100.00		5.0	5.0	200.0				
Mass	RA	Calc. Mass	mDa	PPM	DBE	i-FIT	Norm	Conf(%)	Formula
226.0356	100.00	226.0360	-0.4	-1.8	5.5	295.0	n/a	n/a	C10 H12 N O S2

N-(4-Methoxyphenyl)-1,2-dithiolane-4-carboxamide (2b) ($CDCl_3$)

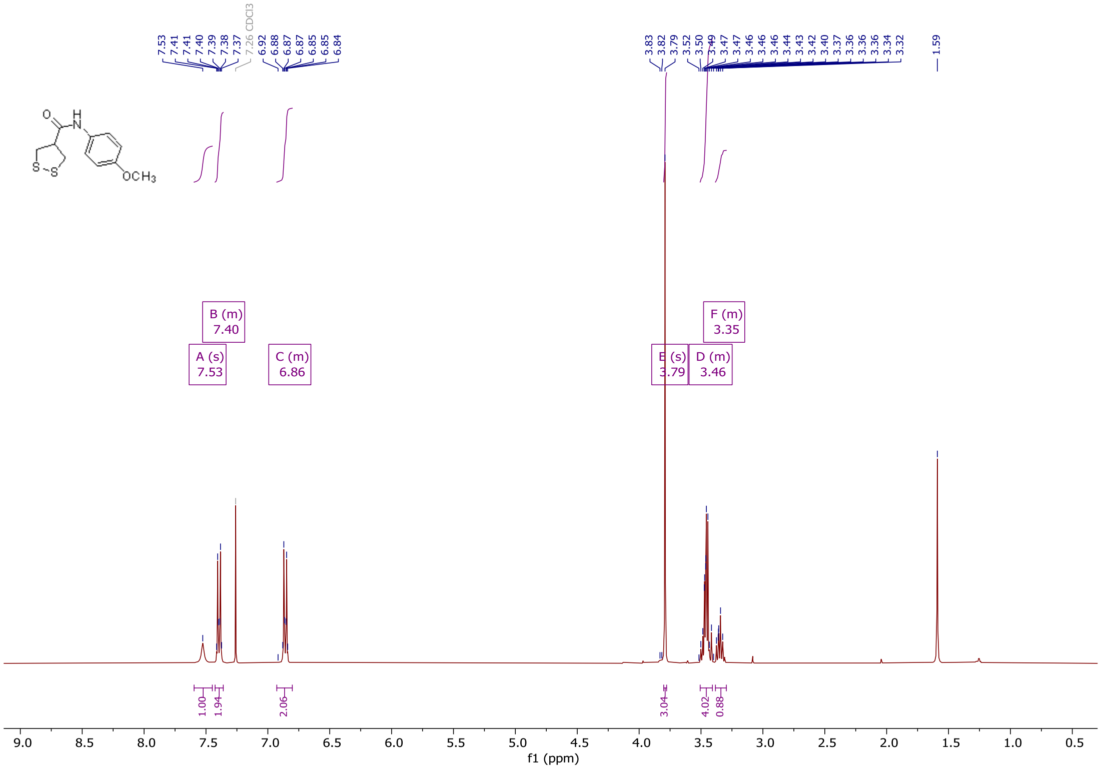

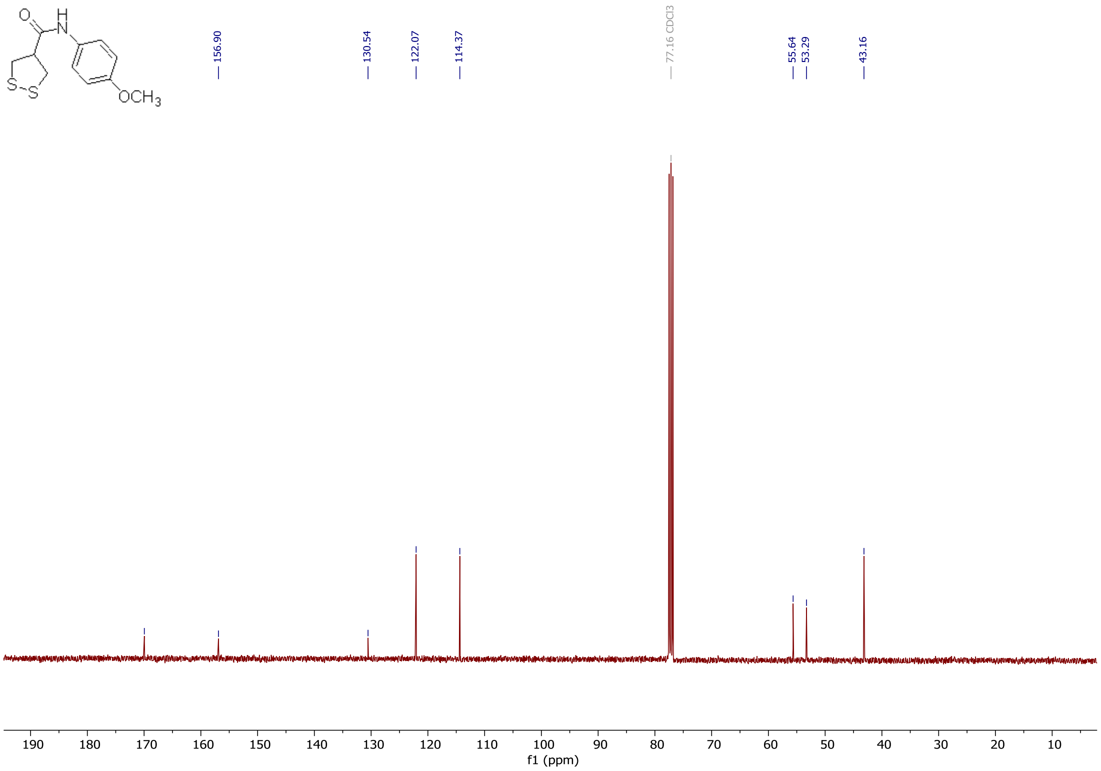

Tolerance = 5.0 PPM / DBE: min = -1.5, max = 200.0
Element prediction: Off
Number of isotope peaks used for i-FIT = 5

Monoisotopic Mass, Even Electron Ions
156 formula(e) evaluated with 1 results within limits (all results (up to 1000) for each mass)
Elements Used:
C: 0-50 H: 0-100 N: 0-3 O: 0-3 S: 0-2

2882 Nikitjuka **2b**

HRMS_2022_04_526 674 (1.929) Cm (673:683-(645:651+699:708))

OSI/FOKL-MS
Synapt G2-Si
1: TOF MS ES+
3.26e+006

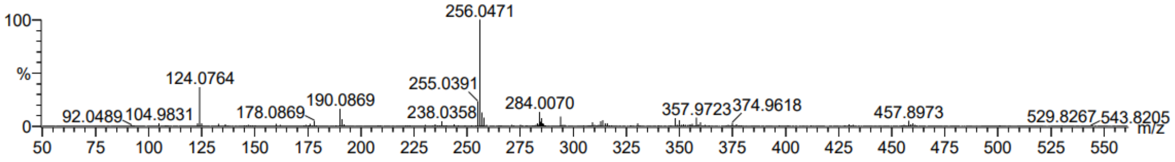

Minimum:	80.00				-1.5				
Maximum:	100.00		5.0	5.0	200.0				
Mass	RA	Calc. Mass	mDa	PPM	DBE	i-FIT	Norm	Conf(%)	Formula
256.0471	100.00	256.0466	0.5	2.0	5.5	633.4	n/a	n/a	C11 H14 N O2 S2

N-(4-(Trifluoromethyl)phenyl)-1,2-dithiolane-4-carboxamide (2c) ($CDCl_3$)

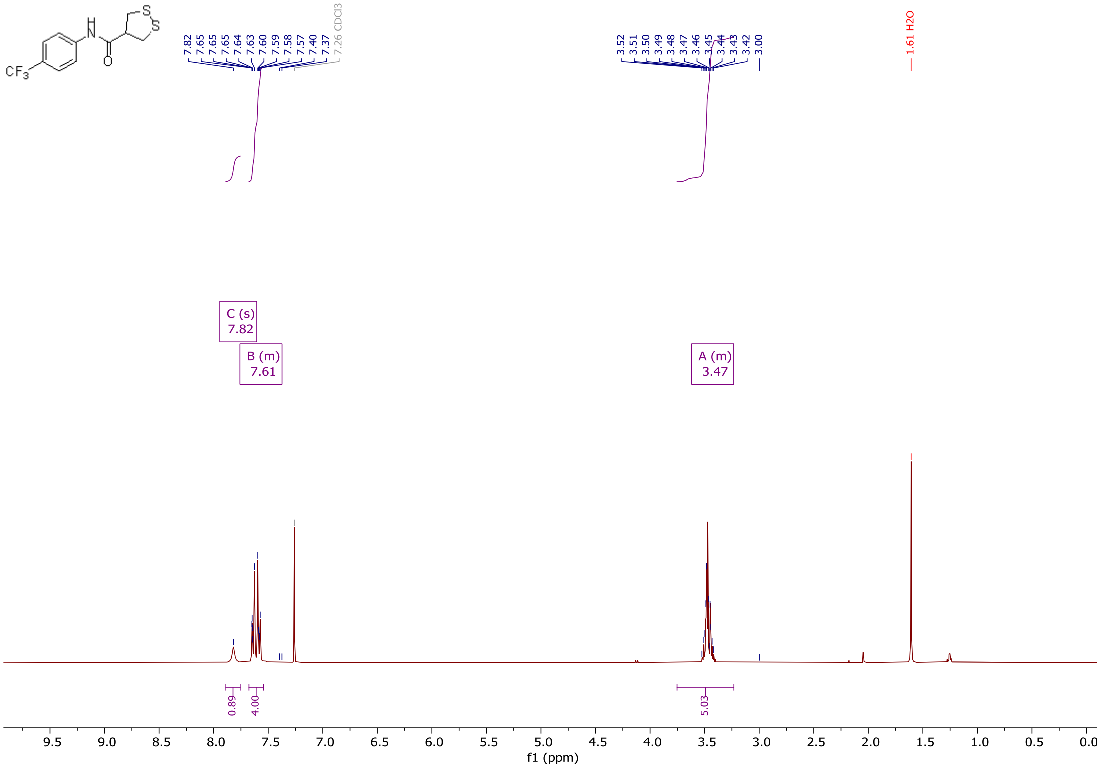

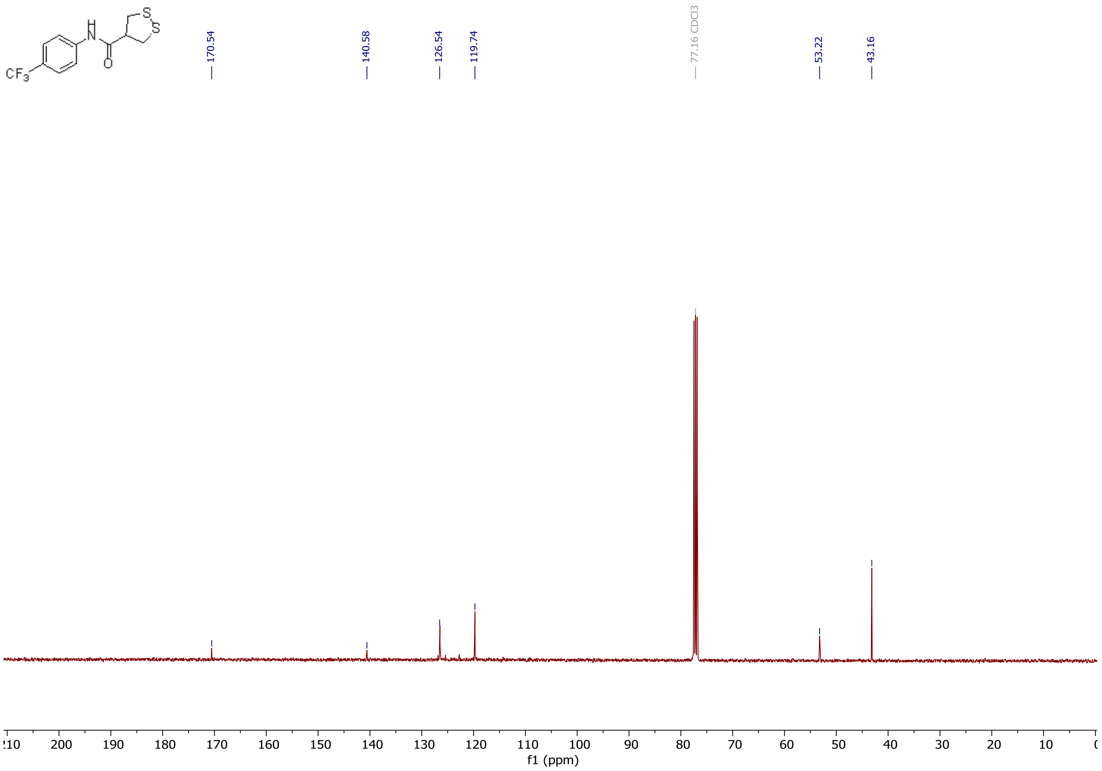

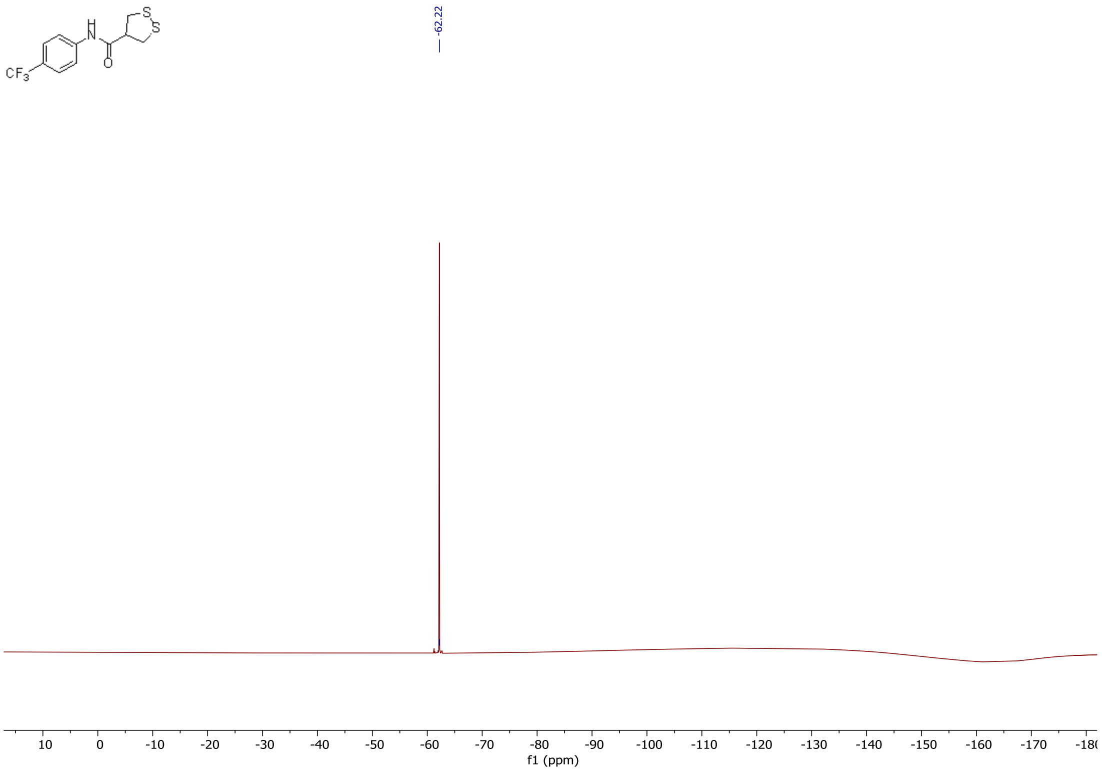

Tolerance = 5.0 PPM / DBE: min = -1.5, max = 200.0
Element prediction: Off
Number of isotope peaks used for i-FIT = 5

2c

Monoisotopic Mass, Even Electron Ions
370 formula(e) evaluated with 1 results within limits (all results (up to 1000) for each mass)
Elements Used:
C: 0-50 H: 0-100 N: 0-2 O: 0-2 F: 0-3 S: 0-2

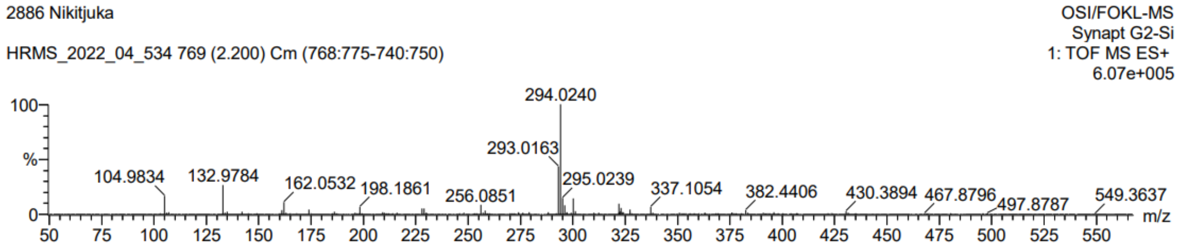

Minimum:	80.00				-1.5				
Maximum:	100.00		2.0	5.0	200.0				
Mass	RA	Calc. Mass	mDa	PPM	DBE	i-FIT	Norm	Conf(%)	Formula
294.0240	100.00	294.0234	0.6	2.0	5.5	513.4	n/a	n/a	C11 H11 N O F3 S2

N-(2,4-dimethoxyphenyl)-1,2-dithiolane-4-carboxamide (2d) ($CDCl_3$)

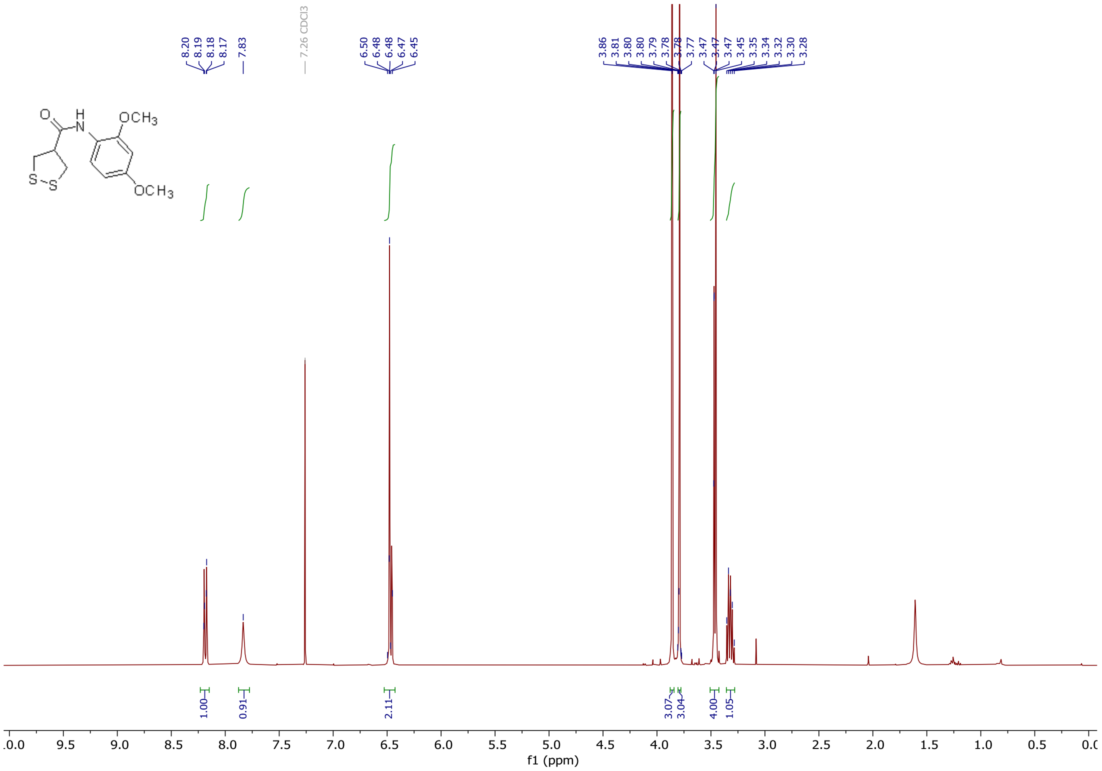

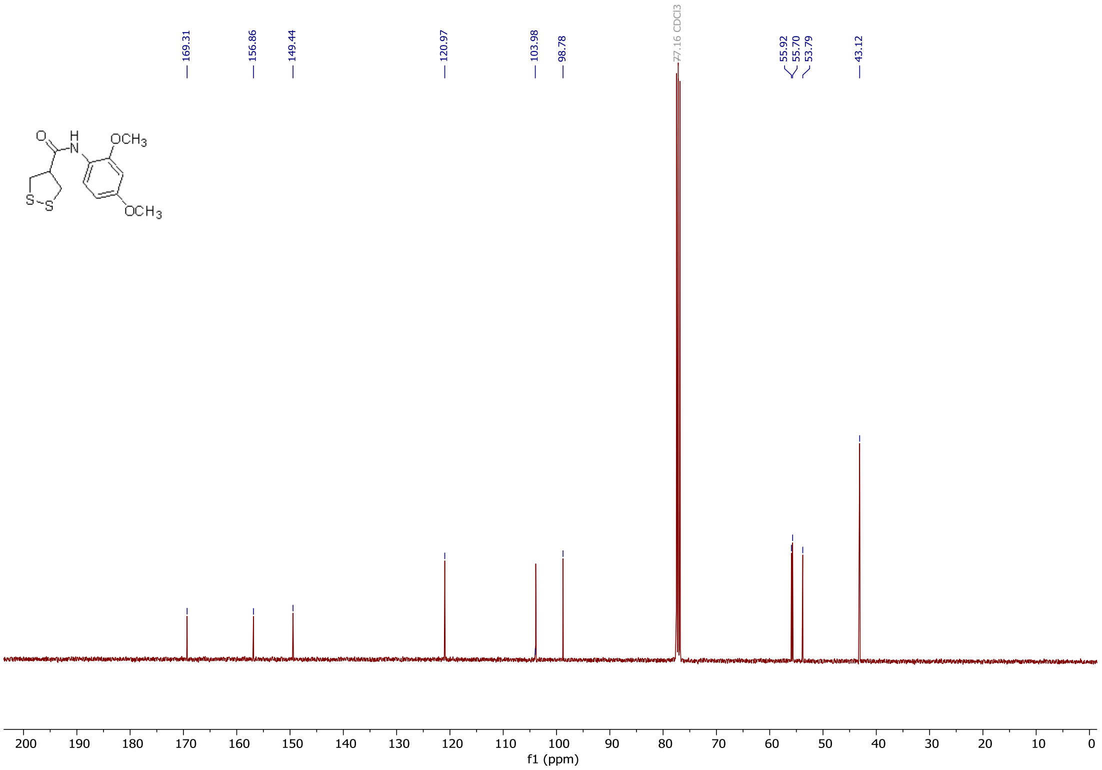

Tolerance = 5.0 PPM / DBE: min = -1.5, max = 200.0
Element prediction: Off
Number of isotope peaks used for i-FIT = 5

Monoisotopic Mass, Even Electron Ions
172 formula(e) evaluated with 1 results within limits (all results (up to 1000) for each mass)
Elements Used:
C: 0-50 H: 0-100 N: 0-3 O: 0-3 S: 0-2

2887 Nikitjuka **2d**

HRMS_2022_04_536 706 (2.017) Cm (706:713-668:678)

OSI/FOKL-MS
Synapt G2-Si
1: TOF MS ES+
7.58e+006

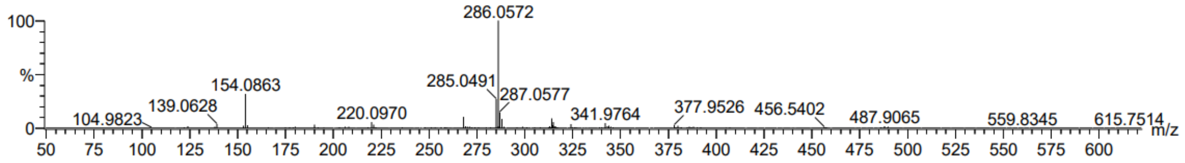

Minimum:	80.00				-1.5				
Maximum:	100.00		2.0	5.0	200.0				
Mass	RA	Calc. Mass	mDa	PPM	DBE	i-FIT	Norm	Conf(%)	Formula
286.0572	100.00	286.0572	0.0	0.0	5.5	894.8	n/a	n/a	C12 H16 N O3 S2

N-(3,5-Dimethoxyphenyl)-1,2-dithiolane-4-carboxamide (2e) (Acetone-d6)

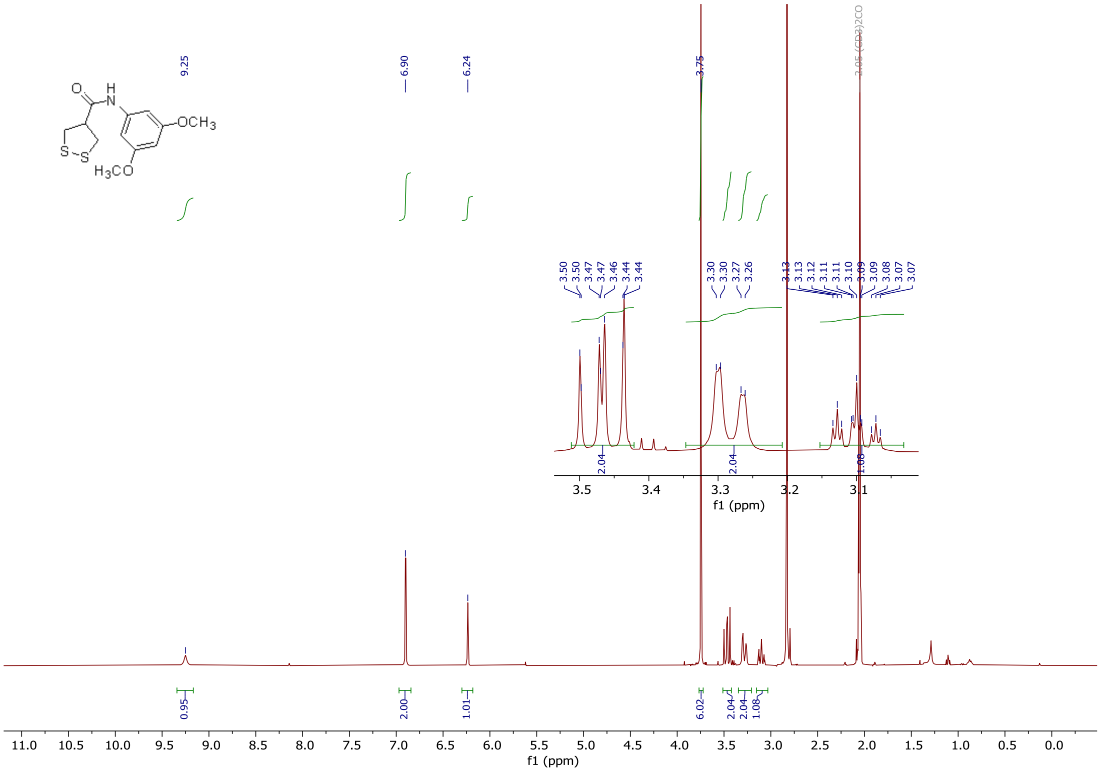

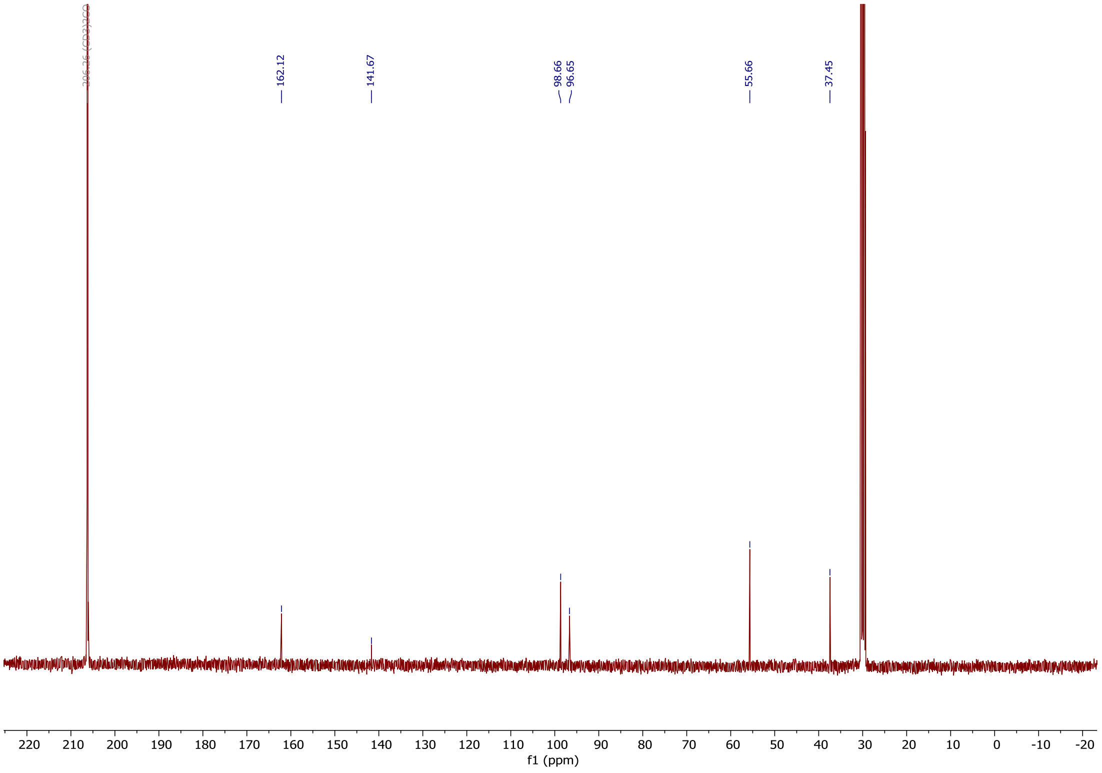

Single Mass Analysis

Tolerance = 3.0 PPM / DBE: min = -0.5, max = 200.0
Element prediction: Off
Number of isotope peaks used for i-FIT = 5

Monoisotopic Mass, Even Electron Ions
552 formula(e) evaluated with 3 results within limits (all results (up to 1000) for each mass)
Elements Used:
C: 1-100 H: 1-150 N: 0-15 O: 0-15 S: 1-2

2576 Nikitjuka **2e**

HRMS_2023_08_080 692 (1.978) Cm (692:701-634:649)

OSI/FOKL-MS
Synapt G2-Si
1: TOF MS ES+
9.01e+006

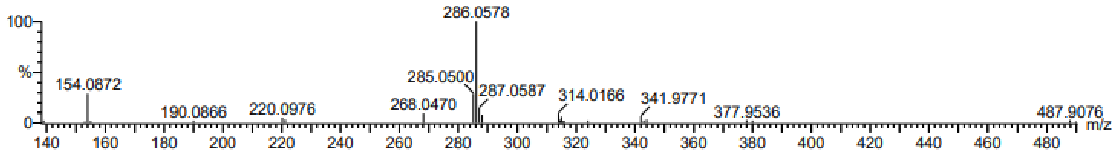

Mass	Calc. Mass	mDa	PPM	DBE	i-FIT	Norm	Conf(%)	Formula
Minimum:				-0.5				
Maximum:		10.0	3.0	200.0				
286.0578	286.0583	-0.5	-1.7	7.5	60.5	0.235	79.09	C5 H8 N11 O2 S
	286.0572	0.6	2.1	5.5	66.9	6.594	0.14	C12 H16 N O3 S2
	286.0570	0.8	2.8	2.5	61.9	1.572	20.77	C4 H12 N7 O6 S

***t*Butyl 2-(1,2-dithiolane-4-carboxamido)-3-methylpentanoate (2f) (CDCl$_3$)**

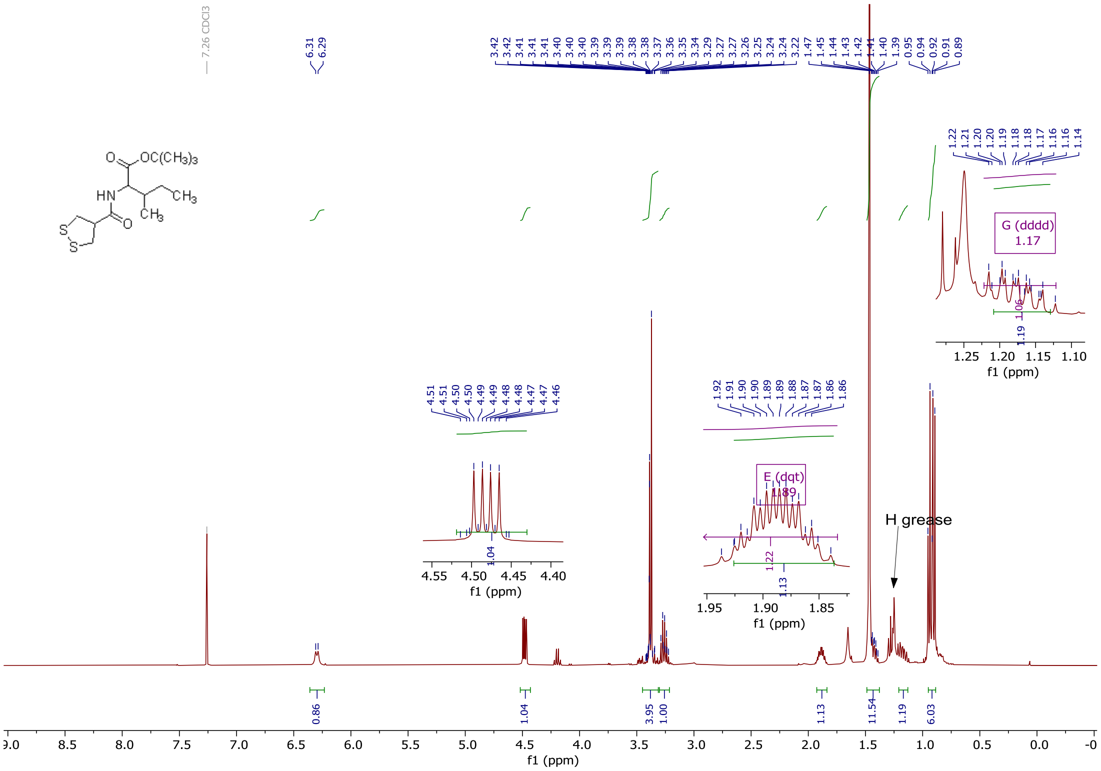

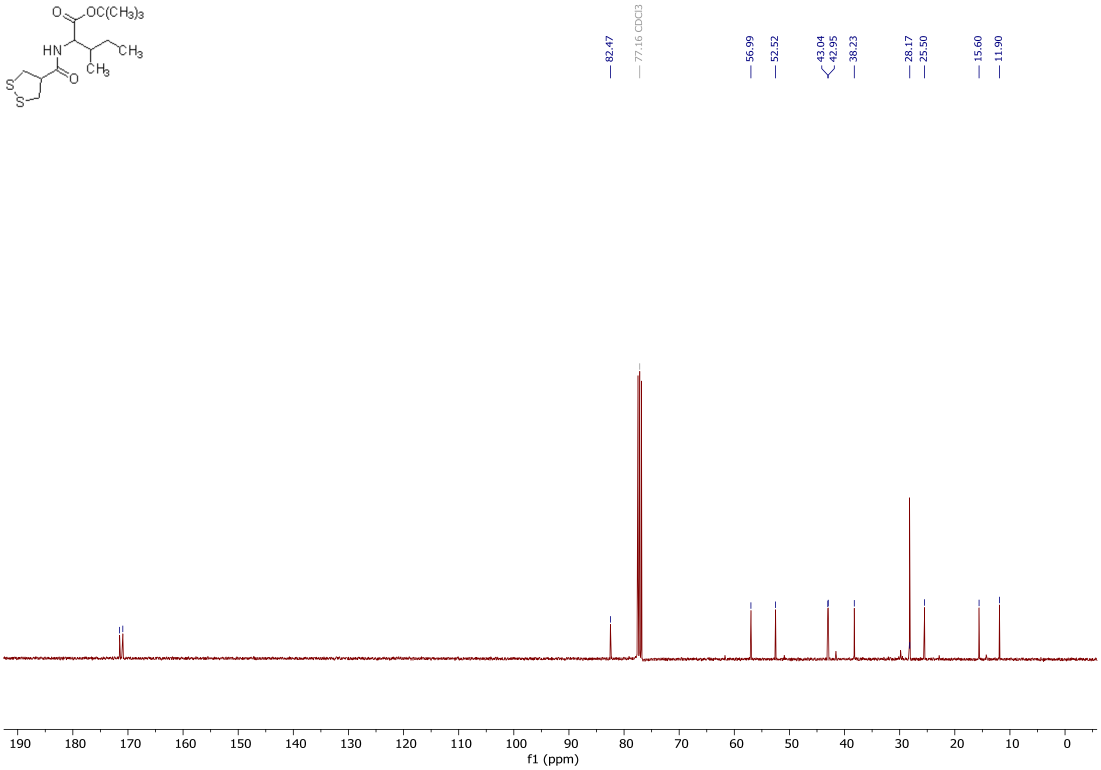

Single Mass Analysis

Tolerance = 5.0 PPM / DBE: min = -1.5, max = 200.0
Element prediction: Off
Number of isotope peaks used for i-FIT = 5

Monoisotopic Mass, Even Electron Ions
188 formula(e) evaluated with 1 results within limits (all results (up to 1000) for each mass)
Elements Used:
C: 0-50 H: 0-100 N: 0-3 O: 0-3 S: 0-2

2888 Nikutjuka. **2f**

HRMS_2022_04_544 804 (2.257) Cm (802:811-777:789)

OSI/FOKL-MS
Synapt G2-Si
1: TOF MS ES-
5.20e+006

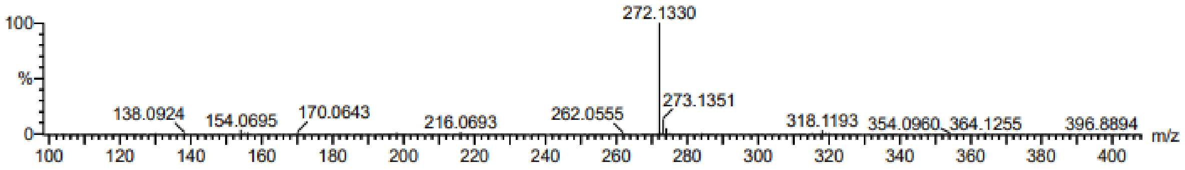

Minimum:				-1.5				
Maximum:		2.0	5.0	200.0				
Mass	Calc. Mass	mDa	PPM	DBE	i-FIT	Norm	Conf(%)	Formula
318.1193	318.1198	-0.5	-1.6	3.5	387.1	n/a	n/a	C14 H24 N O3 S2

2-(1,2-Dithiolane-4-carboxamido)-3-methylpentanoic acid (2g) (MeOH-d4)

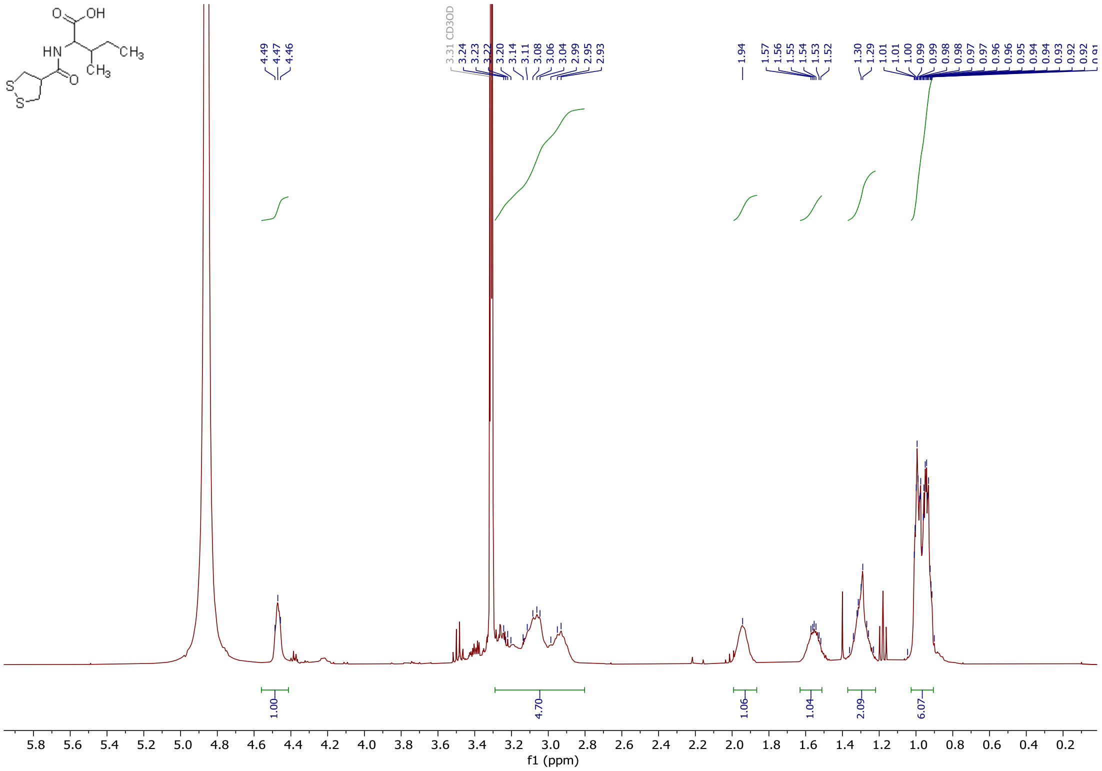

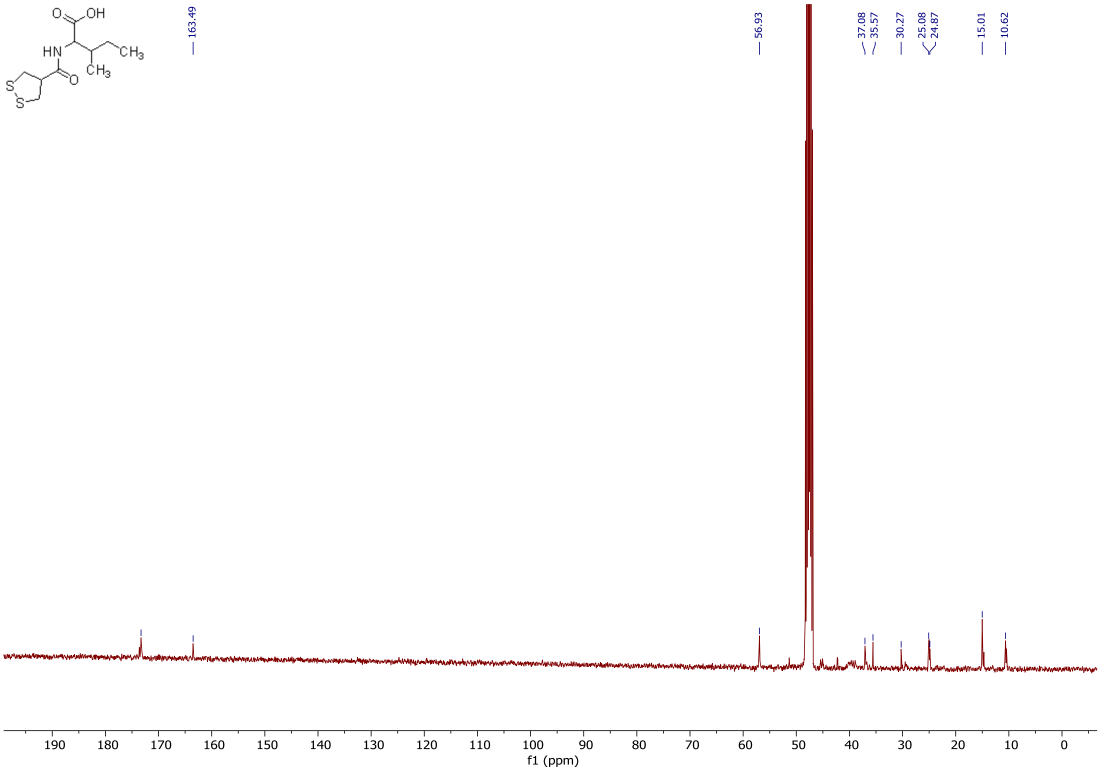

Tolerance = 5.0 PPM / DBE: min = -1.5, max = 200.0
Element prediction: Off
Number of isotope peaks used for i-FIT = 5

Monoisotopic Mass, Even Electron Ions
156 formula(e) evaluated with 1 results within limits (all results (up to 1000) for each mass)
Elements Used:
C: 0-50 H: 0-100 N: 0-3 O: 0-3 S: 0-2

2889 Nikutjuka **2g**

HRMS_2022_04_543 644 (1.814) Cm (642:651-(621:626+679:684))

OSI/FOKL-MS
Synapt G2-Si
1: TOF MS ES-
4.61e+006

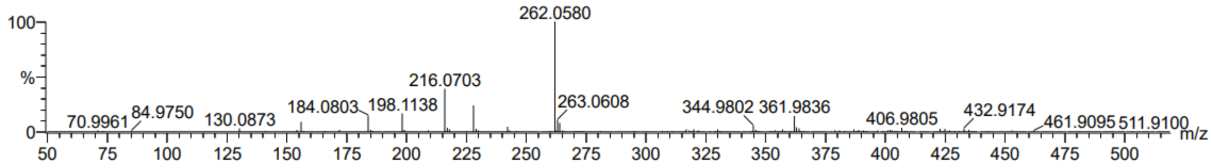

Minimum:	80.00				-1.5				
Maximum:	100.00		2.0	5.0	200.0				
Mass	RA	Calc. Mass	mDa	PPM	DBE	i-FIT	Norm	Conf(%)	Formula
262.0580	100.00	262.0572	0.8	3.1	3.5	799.9	n/a	n/a	C10 H16 N O3 S2

***N*,*N*-Dimethyl-1,2-dithiolane-4-carboxamide (2h)**

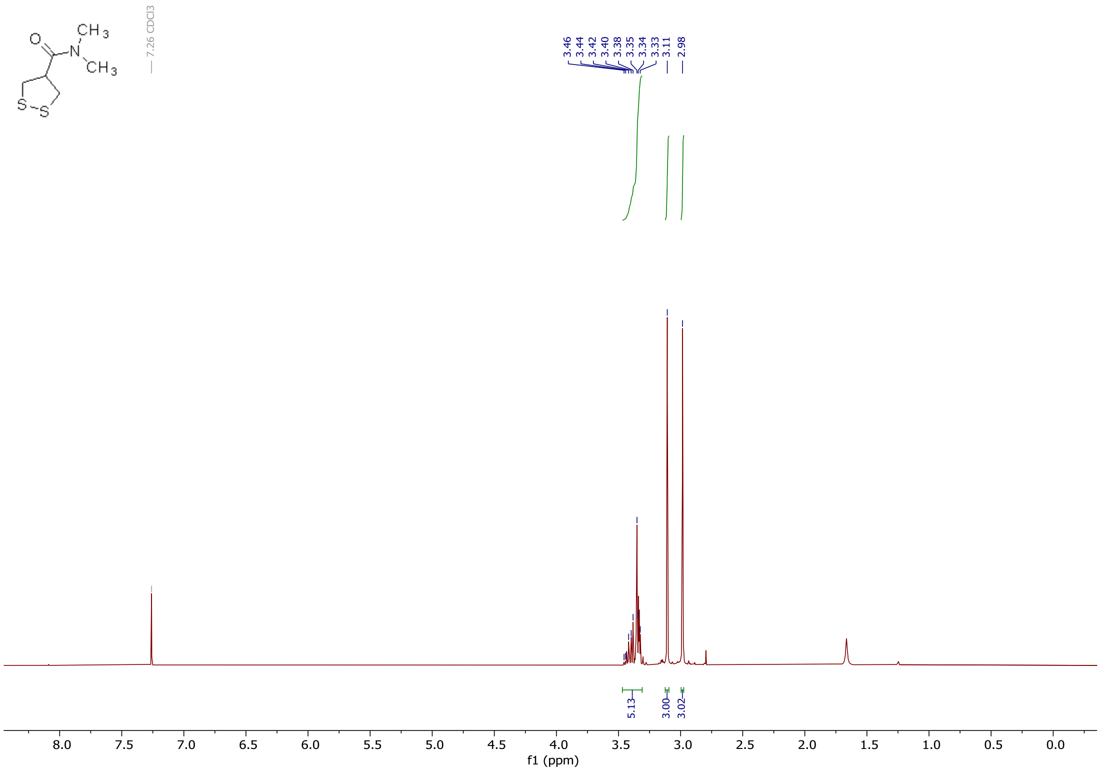

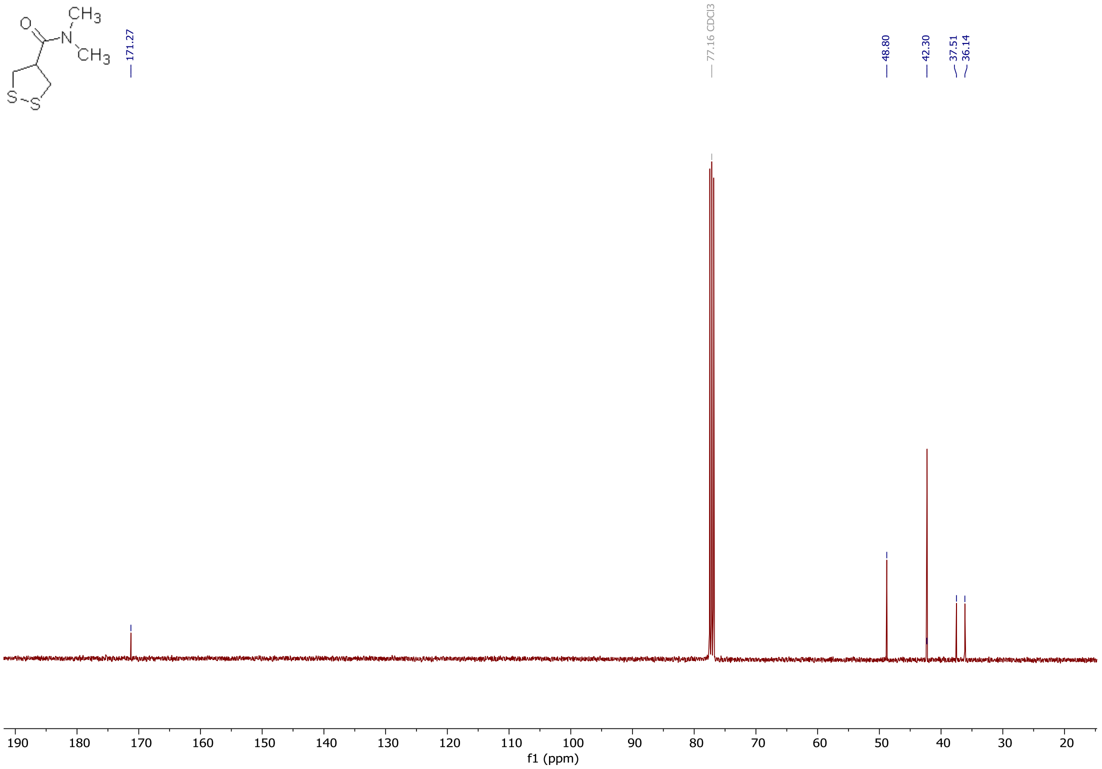

Tolerance = 5.0 PPM / DBE: min = -1.5, max = 200.0
Element prediction: Off
Number of isotope peaks used for i-FIT = 5

Monoisotopic Mass, Even Electron Ions
108 formula(e) evaluated with 1 results within limits (all results (up to 1000) for each mass)
Elements Used:
C: 0-50 H: 0-100 N: 0-3 O: 0-3 S: 0-2

2885 Nikitjuka **2h**

HRMS_2022_04_532 557 (1.598) Cm (557:573-(487:503+603:616))

OSI/FOKL-MS
Synapt G2-Si
1: TOF MS ES+
1.44e+007

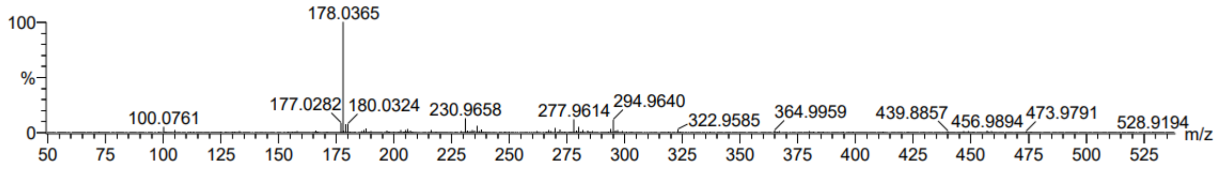

Minimum:	80.00				-1.5				
Maximum:	100.00		5.0	5.0	200.0				
Mass	RA	Calc. Mass	mDa	PPM	DBE	i-FIT	Norm	Conf(%)	Formula
178.0365	100.00	178.0360	0.5	2.8	1.5	919.9	n/a	n/a	C6 H12 N O S2

N-(Methylsulfonyl)-1,2-dithiolane-4-carboxamide (2i) (MeOH-d4)

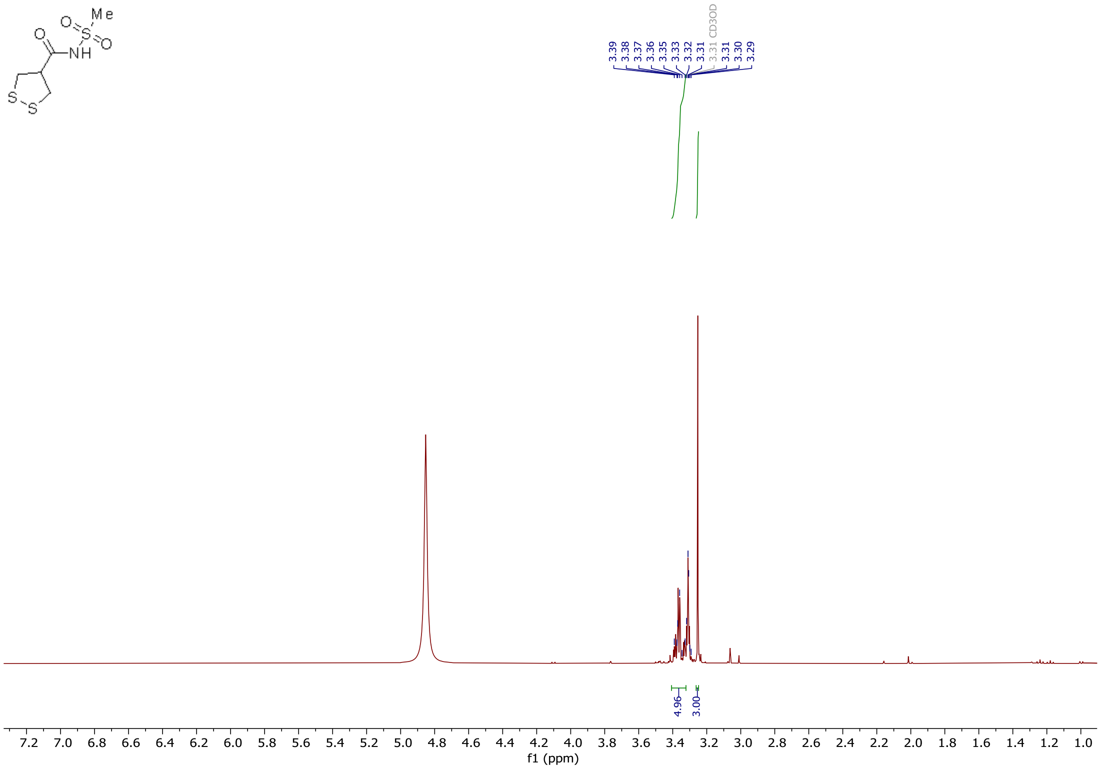

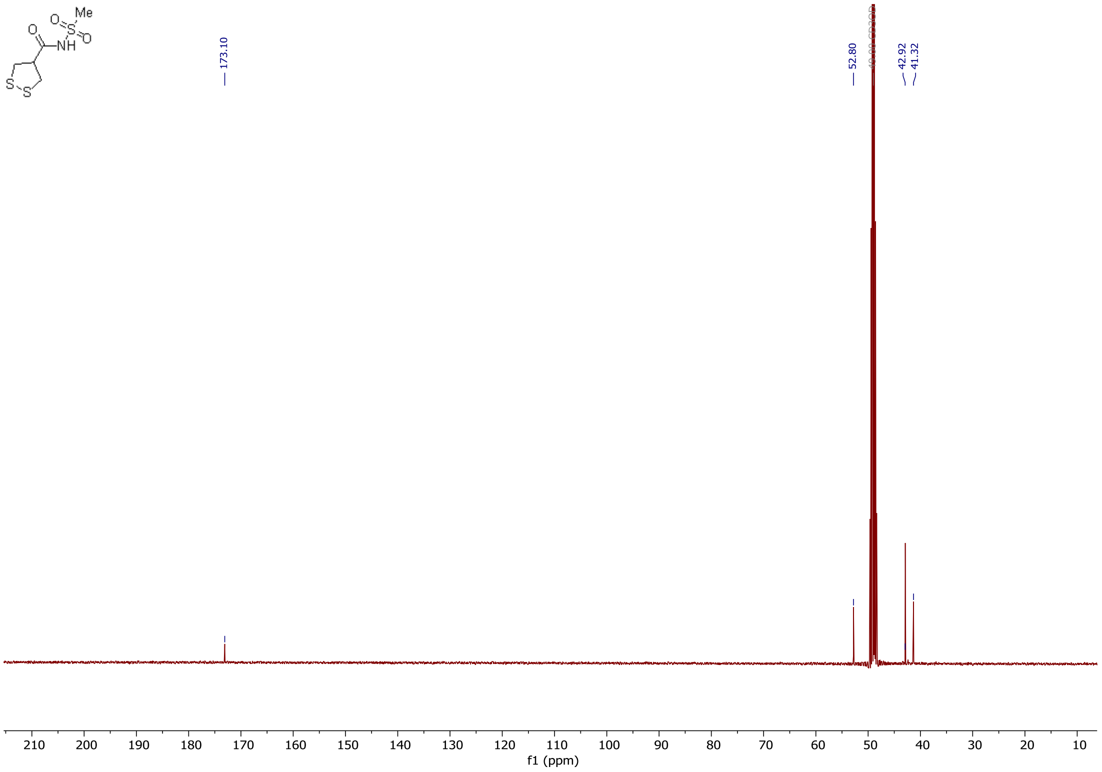

Tolerance = 5.0 PPM / DBE: min = -1.5, max = 200.0
Element prediction: Off
Number of isotope peaks used for i-FIT = 5

Monoisotopic Mass, Even Electron Ions
181 formula(e) evaluated with 1 results within limits (all results (up to 1000) for each mass)
Elements Used:
C: 0-50 H: 0-100 N: 0-2 O: 0-5 S: 0-3

2884 Nikutjuka **2i**

HRMS_2022_04_546 573 (1.614) Cm (573:580-(542:548+599:603))

OSI/FOKL-MS
Synapt G2-Si
1: TOF MS ES-
3.16e+006

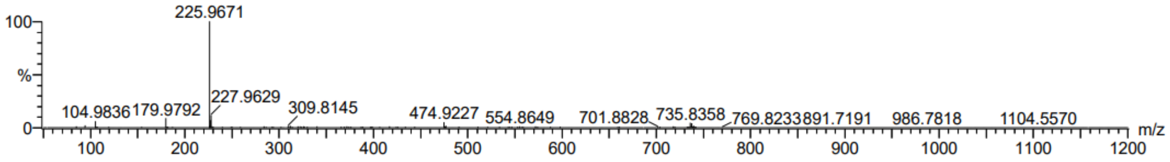

Minimum:	80.00				-1.5				
Maximum:	100.00		2.0	5.0	200.0				
Mass	RA	Calc. Mass	mDa	PPM	DBE	i-FIT	Norm	Conf(%)	Formula
225.9671	100.00	225.9666	0.5	2.2	2.5	718.6	n/a	n/a	C5 H8 N O3 S3

_N_-(2-Oxo-2H-chromen-6-yl)-1,2-dithiolane-4-carboxamide (2j)

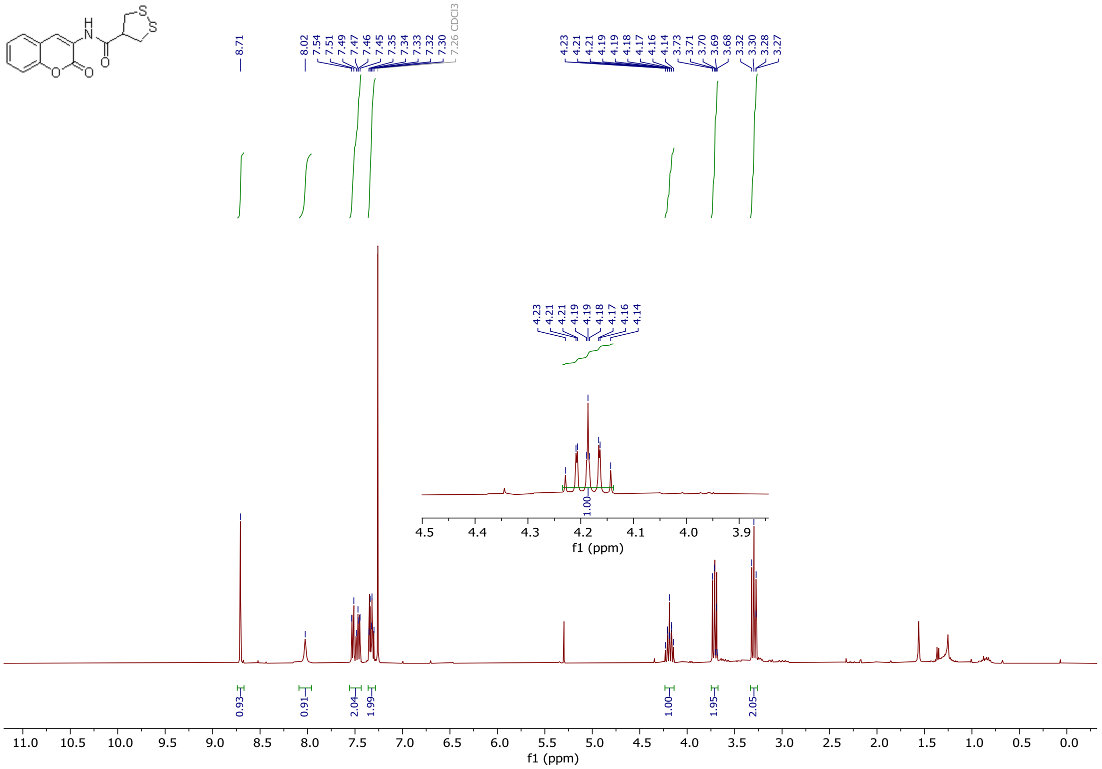

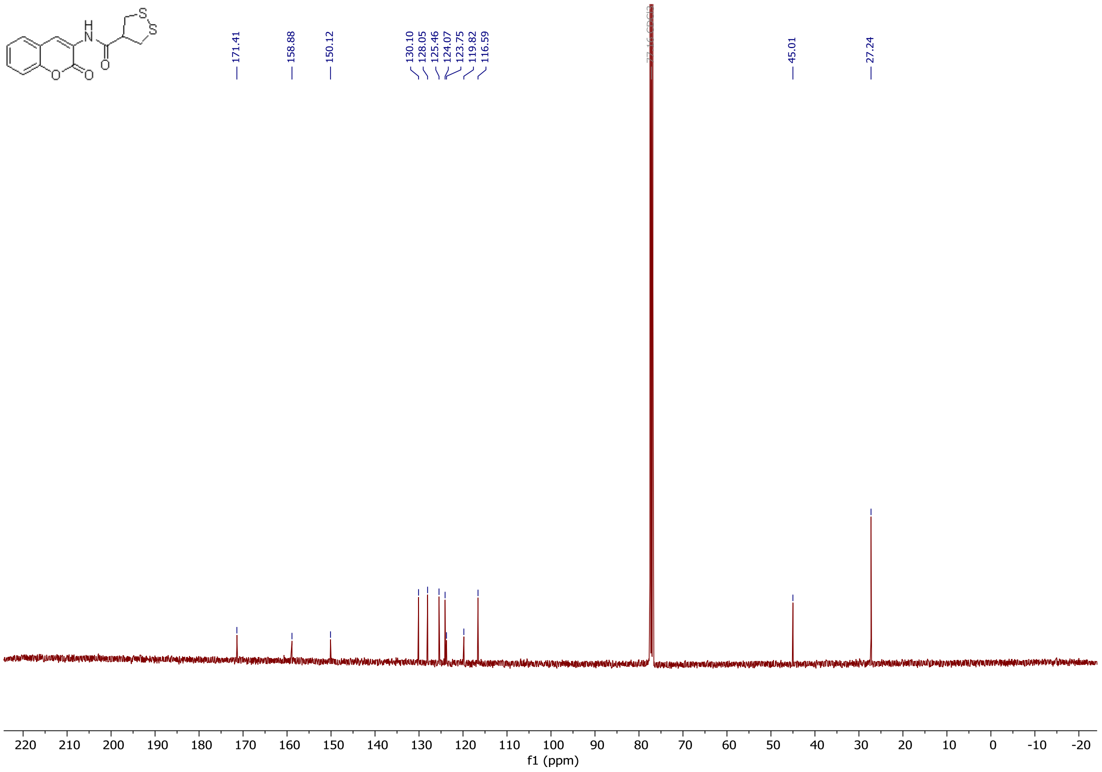

***N*-(2-oxo-2H-chromen-3-yl)-1,2-dithiolane-4-carboxamide (2k) (CD_3CN/Acetone, low solubility)**

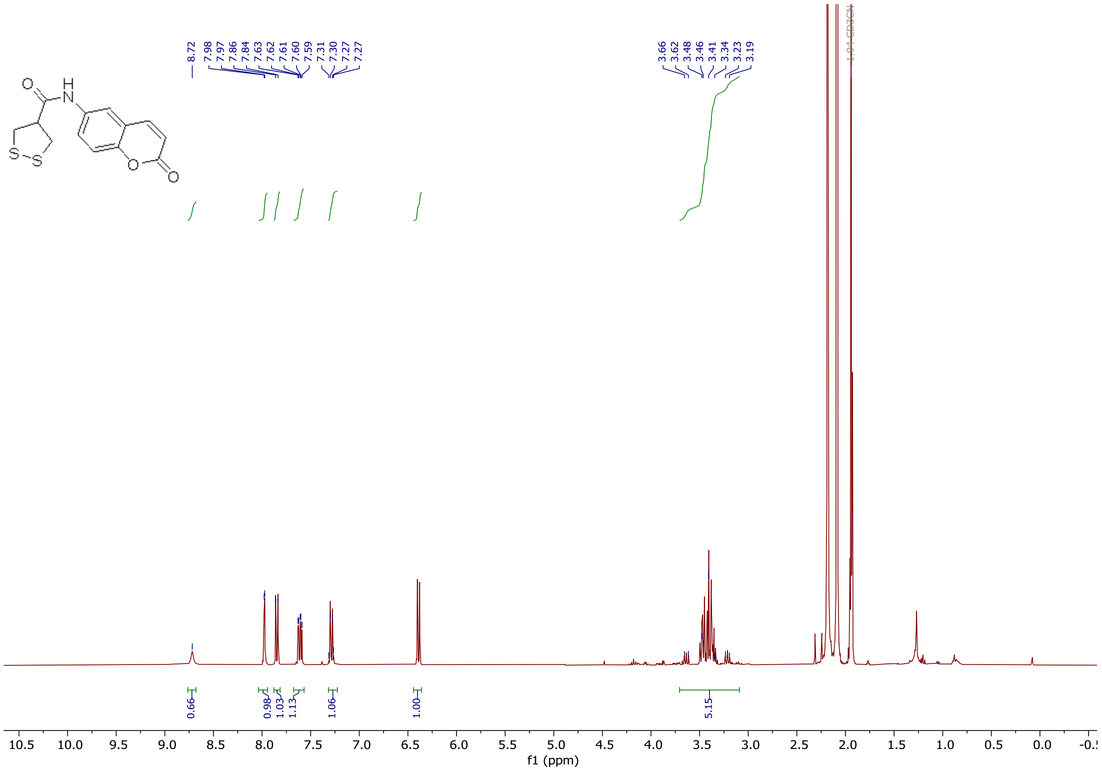

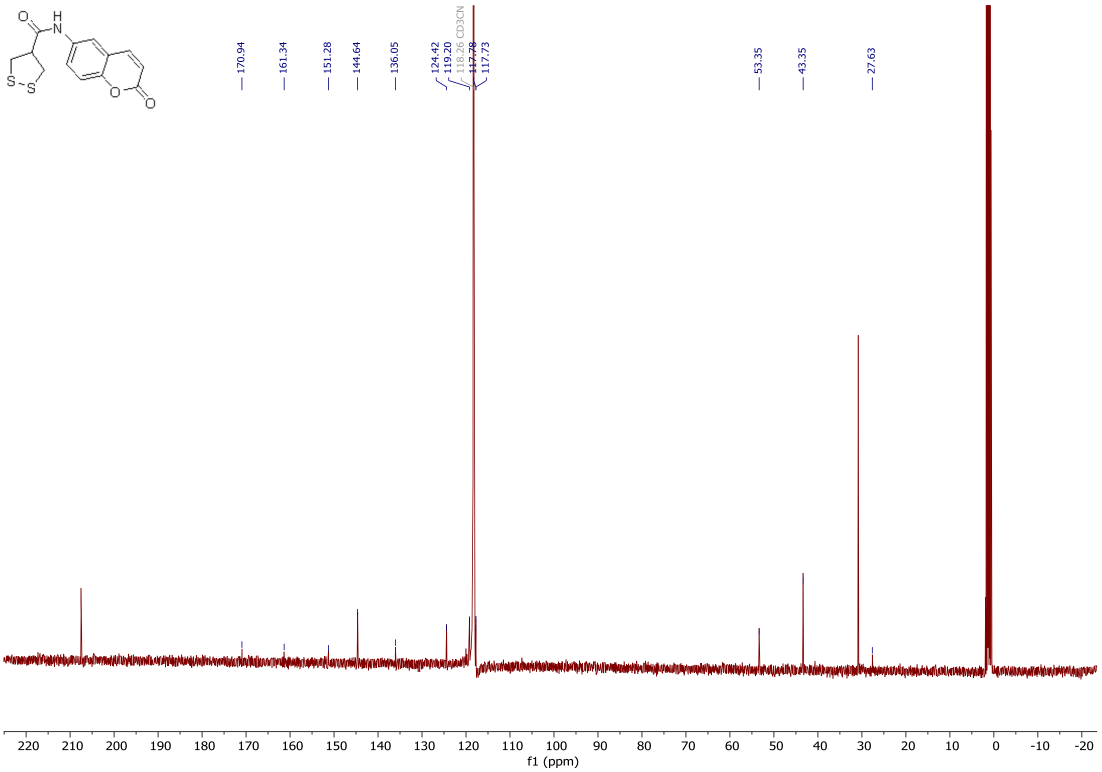

Single Mass Analysis

Tolerance = 5.0 PPM / DBE: min = -1.5, max = 200.0
Element prediction: Off
Number of isotope peaks used for i-FIT = 5

Monoisotopic Mass, Even Electron Ions
248 formula(e) evaluated with 1 results within limits (all results (up to 1000) for each mass)
Elements Used:
C: 0-80 H: 0-150 N: 0-10 O: 0-10 S: 2-2

3118 Nikitjuka. **2k**

OSI/FOKL-MS
Synapt G2-Si
1: TOF MS ES+
7.83e+005

HRMS_2022_05_322 658 (1.885) Cm (658:662-(672:677+629:640))

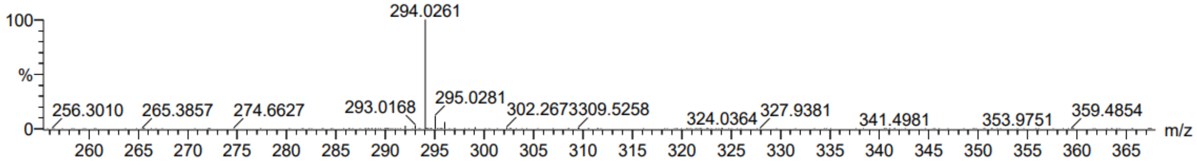

	Calc. Mass	mDa	PPM	DBE	i-FIT	Norm	Conf(%)	Formula
Minimum:				-1.5				
Maximum:		2.0	5.0	200.0				
Mass	Calc. Mass	mDa	PPM	DBE	i-FIT	Norm	Conf(%)	Formula
294.0261	294.0259	0.2	0.7	8.5	178.1	n/a	n/a	C13 H12 N O3 S2

***N*-(4-methyl-2-oxo-2H-chromen-7-yl)-1,2-dithiolane-4-carboxamide (2l, 1,2-dithiolane signal is overlapped with residue water signal)**

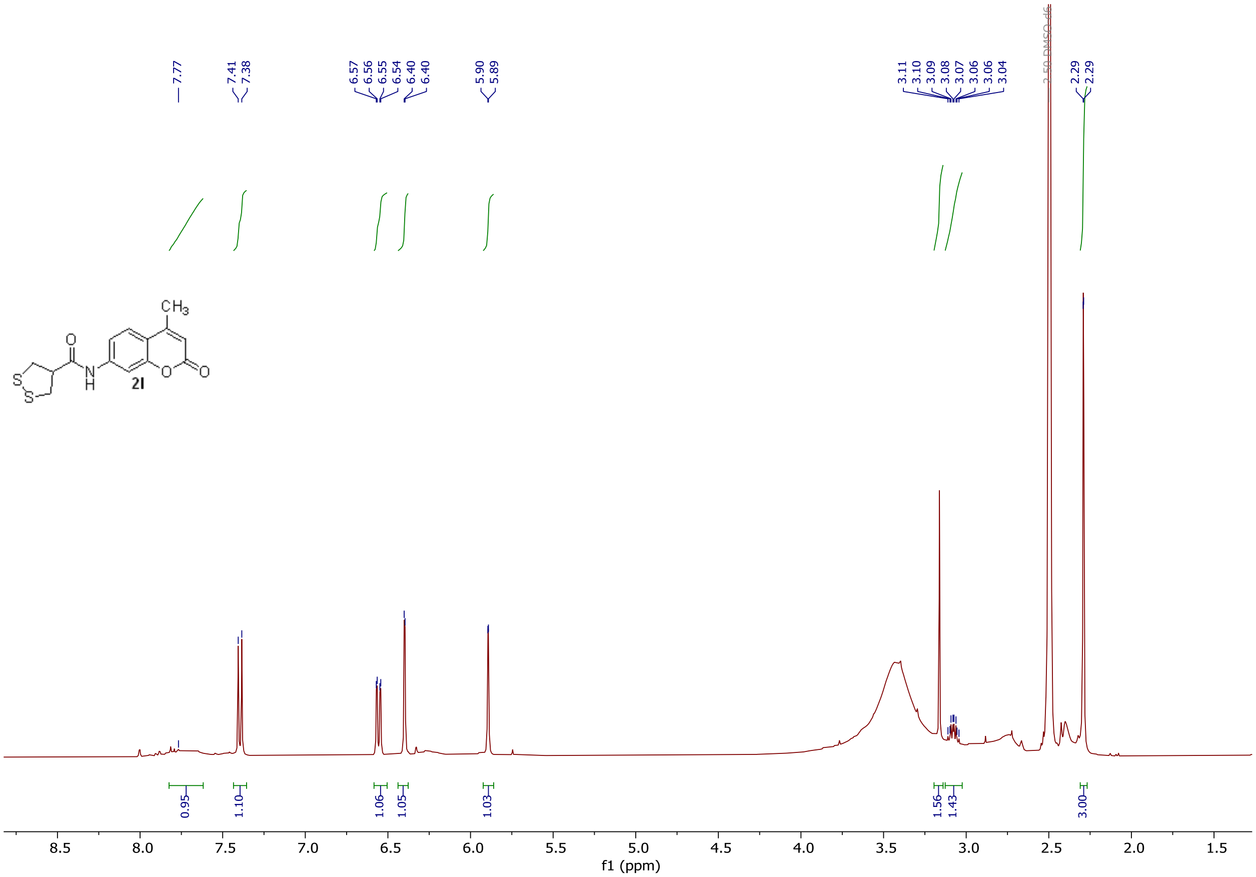

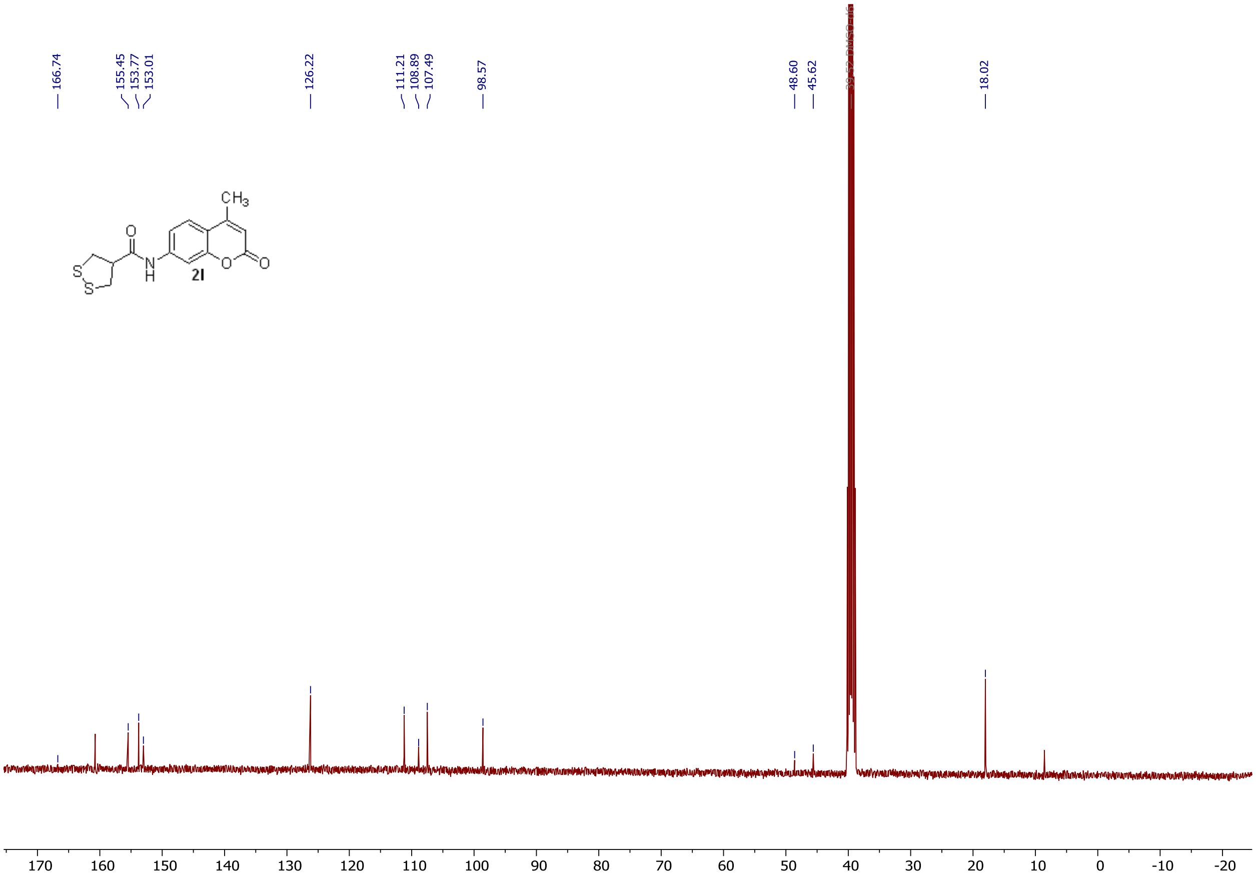

Single Mass Analysis
Tolerance = 5.0 PPM / DBE: min = -1.5, max = 200.0
Element prediction: Off
Number of isotope peaks used for i-FIT = 5

Monoisotopic Mass, Even Electron Ions
276 formula(e) evaluated with 1 results within limits (all results (up to 1000) for each mass)
Elements Used:
C: 0-80 H: 0-150 N: 0-10 O: 0-10 S: 2-2

3119 Nikitjuka **2l**
HRMS_2022_05_324 673 (1.926) Cm (672:679-(654:660+700))

OSI/FOKL-MS
Synapt G2-Si
1: TOF MS ES+
9.46e+005

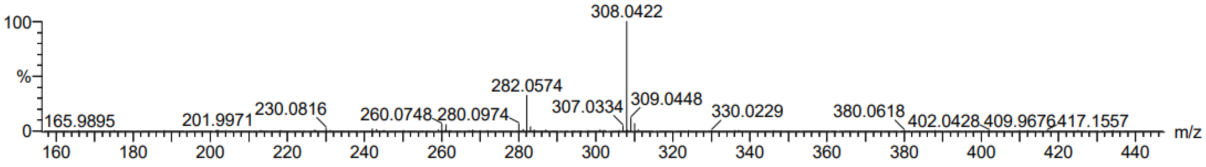

Minimum:				-1.5				
Maximum:		2.0	5.0	200.0				
Mass	Calc. Mass	mDa	PPM	DBE	i-FIT	Norm	Conf(%)	Formula
308.0422	308.0415	0.7	2.3	8.5	694.8	n/a	n/a	C14 H14 N O3 S2

Determination of the Reaction Rate and IC_{50} of colorimetric TrxR1 enzymatic assays

IC_{50} was obtained using Thioredoxin Reductase Colorimetric Assay Kit provided by Cayman Chemicals and the provided kit booklet was used for the IC_{50} calculation (Thioredoxin Reductase Colorimetric Assay Kit (TrxR, TxnR) | Cayman Chemical). In brief, the reaction rate (ΔA) was determined and corrected:

ΔA/min(sample) – [ΔA/min(sample+enzyme) – DA/min(Background+enzyme)]

$$\text{TrxR activity} \quad \frac{\Delta A}{\quad}$$

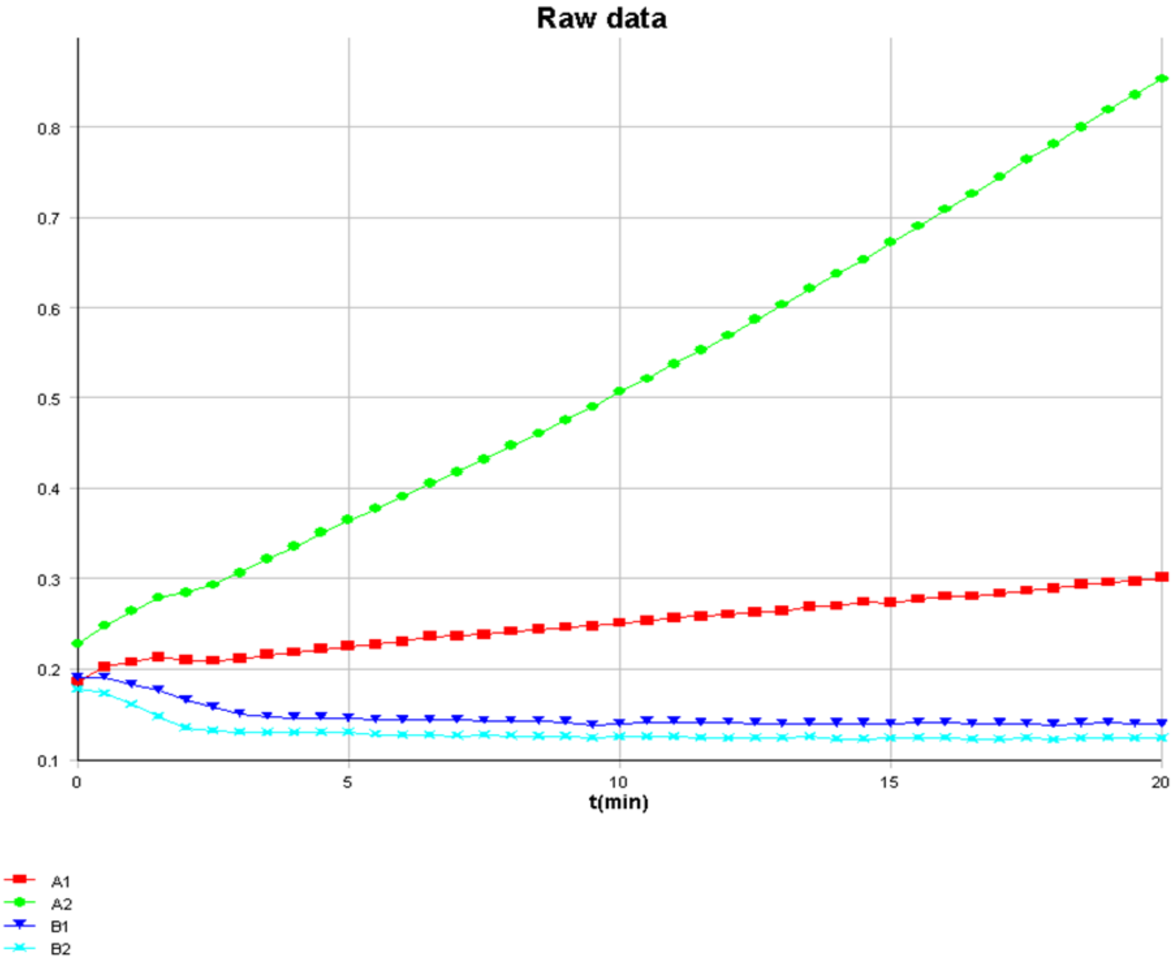

Figure S1. Representative example of activity plot of the rat liver TrxR1 (x = time (minutes); y = absorbance at 405 nm).
